# Thermal block of action potentials is primarily due to voltage-dependent potassium currents: a modeling study

**DOI:** 10.1088/1741-2552/ab131b

**Published:** 2019-03-25

**Authors:** Mohit Ganguly, Michael W Jenkins, E Duco Jansen, Hillel J Chiel

**Affiliations:** 1 Department of Biomedical Engineering, Vanderbilt University, Nashville, TN, United States of America; 2 Biophotonics Center, Vanderbilt University, Nashville, TN, United States of America; 3 Department of Pediatrics, Case Western Reserve University, Cleveland, OH, United States of America; 4 Department of Biomedical Engineering, Case Western Reserve University, Cleveland, OH, United States of America; 5 Department of Biology, Case Western Reserve University, Cleveland, OH, United States of America; 6 Department of Neurosciences, Case Western Reserve University, Cleveland, OH, United States of America

**Keywords:** thermal block, infrared neural inhibition, computational model, unmyelinated axons, voltage-gated potassium channels, squid giant axon, scaling

## Abstract

**Objective.:**

Thermal block of action potential conduction using infrared lasers is a new modality for manipulating neural activity. It could be used for analysis of the nervous system and for therapeutic applications. We sought to understand the mechanisms of thermal block.

**Approach.:**

To analyze the mechanisms of thermal block, we studied both the original Hodgkin/Huxley model, and a version modified to more accurately match experimental data on thermal responses in the squid giant axon.

**Main results.:**

Both the original and modified models suggested that thermal block, especially at higher temperatures, is primarily due to a *depolarization-activated hyperpolarization* as increased temperature leads to faster activation of voltage-gated potassium ion channels. The minimum length needed to block an axon scaled with the square root of the axon’s diameter.

**Significance.:**

The results suggest that voltage-dependent potassium ion channels play a major role in thermal block, and that relatively short lengths of axon could be thermally manipulated to selectively block fine, unmyelinated axons, such as C fibers, that carry pain and other sensory information.

## Introduction

Targeted optical manipulation of the nervous system has become an exciting new possibility in recent years. Using lasers, it is possible to deliver infrared light to tissue with high spatial and temporal specificity to control excitable tissues such as the sciatic nerve (Wells et al 2005), visual cortex (Cayce et al 2014), cochlear neurons (Matic et al 2013), and both embryonic and adult hearts (Jenkins et al 2010, 2013). The new field of infrared control of excitable tissue has been recently reviewed (Thompson et al 2014). Several possible mechanisms have been suggested for IR-induced excitation, including the induction of capacitive currents due to thermal gradients (Shapiro et al 2012, Plaksin et al 2017), activation of mitochondrial calcium currents (Dittami et al 2011, Lumbreras et al 2014), endoplasmic reticulum (Tolstykh et al 2017), and direct actions on ion channels (Albert et al 2012).

More recently, it has been shown that infrared laser light can be used to inhibit both action potentials traveling through axons and through cardiomyocytes (Duke et al 2013, Lothet et al 2014, Wang et al 2016, Lothet et al 2017). Rather than inducing a thermal gradient, it appears that the inhibitory mechanism is due to raising tissue temperature (Duke et al 2013). Thus, understanding the effects of temperature on axonal conduction could be very useful for more precisely designing ways of controlling neural activity.

A recent study demonstrated that IR could selectively block small-diameter axons prior to blocking large-diameter axons, both in an invertebrate (the marine mollusk *Aplysia californica*) and in a mammal (the vagus nerve of the musk shrew *Suncus murinus*) (Lothet et al 2017). Since electrical stimulation or inhibition generally affect large diameter axons before affecting small diameter axons (Rattay 1986), a modality such as IR that selectively controls small-diameter axons could be the basis for new ways of analyzing neuronal function and could lead to novel therapeutic interventions.

Since IR inhibition was demonstrated in both invertebrate and vertebrate axons, we sought to analyze the potential mechanism of thermal inhibition by using computational models of a well-studied invertebrate model, the squid giant axon (Hodgkin and Huxley 1952). We chose to focus on the squid giant axon because, to our knowledge, it is the only experimental system in which there is both a biophysically-based model of action potential generation and propagation, and experimental data about the effects of temperature on characteristics of the action potential (i.e. rates of rise, rates of fall, changes in axial resistance) at the level of a single axon (Rosenthal and Bezanilla 2000, 2002).

A mechanism of thermal inhibition had previously been proposed. Initial studies of the effects of temperature on the generation of the action potential (Hodgkin and Katz 1949) led Huxley to postulate that, at higher temperatures, potassium currents overwhelm sodium currents, leading to action potential failure (Huxley 1959). Other temperature-dependent changes, however, could account for thermal inhibition. Rosenthal and Bezanilla studied the responses of the giant axon in squid acclimated to seasonally warm or cold temperatures (Rosenthal and Bezanilla 2000) and in squid living in temperate or tropical climates (Rosenthal and Bezanilla 2002). Their studies suggested that in addition to changes in the kinetics of the voltage-gated ion channels, there may be changes in axial resistance with temperature. Other studies suggested that the sodium/potassium pump (Carpenter and Alving 1968) and even the peak conductances of the voltage-gated channels (Cao and Oertel 2005) could change with temperature.

Thus, we sought to test Huxley’s hypothesis that activation of voltage-dependent potassium ion channels was primarily responsible for thermal inhibition. In the original Hodgkin Huxley model, the only mechanism by which temperature affected the model was through the temperature-dependence of the gating variables—m and h for the voltage-dependent sodium ion channels, and n for the voltage-dependent potassium ion channels. Would the same mechanism that Huxley had proposed operate in a modified model that more accurately captured the actual experimentally measured responses to temperature, and that incorporated changes in peak ion channel conductances, axial resistance, and the sodium/potassim pump? By addressing this question, we could explore a much larger set of temperature-dependent models than the original Hodgkin Huxley model. We therefore modified the original model to allow it to more accurately capture the experimental data, and tested whether the original mechanism proposed by Huxley primarily accounted for thermal block. We then sought to determine the effect of scaling of axon diameter to see the applicability of these results to small-diameter unmyelinated axons, such as vertebrate C fibers.

The results do support Huxley’s hypothesis that thermally-induced block, especially at higher temperatures, is primarily due to increased activation of voltage-dependent potassium ion channels in response to depolarization. In response to the depolarizing currents from an advancing action potential, the membrane depolarizes. This activates the voltage-dependent potassium ion channels, allowing potassium ions to flow out of the neuron, which in turn hyperpolarizes the neuron. The hyperpolarizing current through these channels antagonizes the depolarizing current that activated them. Thus, this mechanism effectively and rapidly blocks the propagation of the action potential. Note that a hyperpolarizing current is more effective than simply blocking all the ion channels, since a depolarizing current from an advancing action potential would propagate through the passive region of blocked ion channels, diminishing only as it leaked out through the passive components of the axonal membrane (i.e. the leak channels and the capacitance), rather than being actively antagonized.

## Methods

### Model axon geometry

The squid giant axon varies in size, but we chose a standard length of 100 mm and diameter of 0.5 mm (500 *μ*m) based on values reported in the literature (Rosenthal and Bezanilla 2000). We implemented this geometry in the NEURON simulation environment (Carnevale and Hines 2006). In general, the differential equations for the model (see below) were numerically integrated using a time step of 0.01 ms, and the voltages and ionic currents were measured in each segment of the model neuron. For studies in which compensatory current was injected to maintain the resting potential (see below), the time step was reduced considerably (to 0.2 *μ*s) to ensure high accuracy.

To ensure stability of all state variables, the system was simulated for a total of 500 ms, and stimulating current was injected into the model after 250 ms. Depolarizing current (1 ms duration) was injected within the first segment of the first section of the model axons to initiate an action potential. For model axons of diameter greater than 10 *μ*m, 2000 nA of depolarizing current was used. For model axons of diameter less than 10 *μ*m, 100 nA of depolarizing current was used.

To explore how axon diameter affects its response to temperature, we varied axon diameters from 0.5 *μ*m to 500 *μ*m. The axon was divided into either two or three sections, each having different lengths and properties, i.e. temperature, resistivity, and ionic conductances (as described below). The number of segments within each section was chosen to allow for precise measurements of threshold block lengths over the entire length of the model axon. In the modified Hodgkin/Huxley model, the axial resistances varied with temperature. Thus, to construct regions of different temperature, we used the built-in NEURON function *connect* to ensure that each region had the appropriate axial resistance for its temperature.

Segmental currents over the j different segments (where j ranged from 1 to the total number of segments) were computed as

(1) $$I_{\text{segment}}(j)=\frac{V_{j-1}-V_{j}}{r_{a,j-1,j}}-\frac{V_{j}-V_{j+1}}{r_{a,j,j+1}},$$

where $V_{j-1}$ corresponded to the voltage of the previous segment (if there was a previous segment), $V_{j+1}$ corresponded to the voltage of the next segment (if there was a next segment), $r_{a,j-1,j}$ corresponded to the axial resistance between compartment j−1 and j, and $r_{a,j,j+1}$ corresponded to the axial resistance between compartment j and j+1. Note that $r_{a}=\frac{R_{a}}{\pi d^{2}/4}*L$, where $r_{a}$ is the axial resistance, $R_{a}$ is the axial resistivity (in Ω cm), L is the length of the segment in μm, d is the axon diameter (in μm), and $r_{a}$’s units of Ω cm μm ${\mu \text{m}}^{-2}$ yield 10^4^ Ω. Multiplying this by 10^−2^ makes it possible to report the resistance in MΩ.

### Simulating changing temperature in the model

Temperature changes were applied to the model neurons in two ways. First, to determine the changes in currents that occur as an action potential encounters a change in temperature, we maintained one half of a model axon at the base temperature of 6.3 °C while varying the temperature of the second half of the axon. For these studies, the model axon consisted of two sections. By increasing the temperature of half of the axon (the second section), we ensured that transient currents induced by the initiation of an action potential, which occur at the beginning of the first section, were minimized. In the NEURON simulation environment, temperature is a global variable that is uniformly applied to every segment. We changed temperature to be a local variable so that we could access and modify the temperature of each segment independently. Ordinarily, NEURON uses the TABLE command in the .mod file to access a look-up table for all segments; by commenting out this command in the .mod file, the NEURON program evaluated the differential equations at every segment, rather than using the default parameters in the lookup table to decrease run time. This usually increased the simulation duration by a factor of about five.

To approximate a continuously changing temperature profile that might be induced by a thermal source such as a laser, we calculated a temperature profile, T(x), that interpolated smoothly between an initial temperature $T_{1}$ (6.3 °C) and a final temperature $T_{2}$ (25 °C) at all points x along the axon, such that the temperature began to change from its initial value $T_{1}$ at location a along the axon (10 mm before the midpoint, or 40 mm from the beginning of the axon) and reached temperature value $T_{2}$ at location b along the axon (10 mm after the midpoint, or 60 mm after the beginning of the axon). The temperature reached an intermediate temperature value of $\left(T_{2}+T_{1}\right)/2$ at the midpoint between points a and b, i.e. at (a+b)/2:

(2) $$T(x)=T_{1}+\left(T_{2}-T_{1}\right)F(x,a,b),$$

(3) $$F(x,a,b)=\{\begin{array}{cc} 0, & x⩽a,\\ 2{\left(\frac{x-a}{b-a}\right)}^{2}, & a<x⩽\frac{\left(a+b\right)}{2},\\ 1-2{\left(\frac{x-b}{b-a}\right)}^{2}, & \frac{\left(a+b\right)}{2}<x⩽b\\ 1, & x⩾b. \end{array}$$

Second, temperature change was applied along the central region of a model axon to determine the minimum length of elevated temperature that could block action potential conduction. The axon was modeled using three sections, and the length of the central section that was subjected to elevated temperatures was systematically varied until the action potential no longer propagated beyond the region of block. We classified an action potential as blocked if the potential at the end of the axon never exceeded −60 mV, which is far below the threshold for initiating an action potential. Note that the resting potential of the model is −65 mV, so that this is a fairly conservative criterion.

### Temperature-dependent parameters and functions

**Figure 1.**
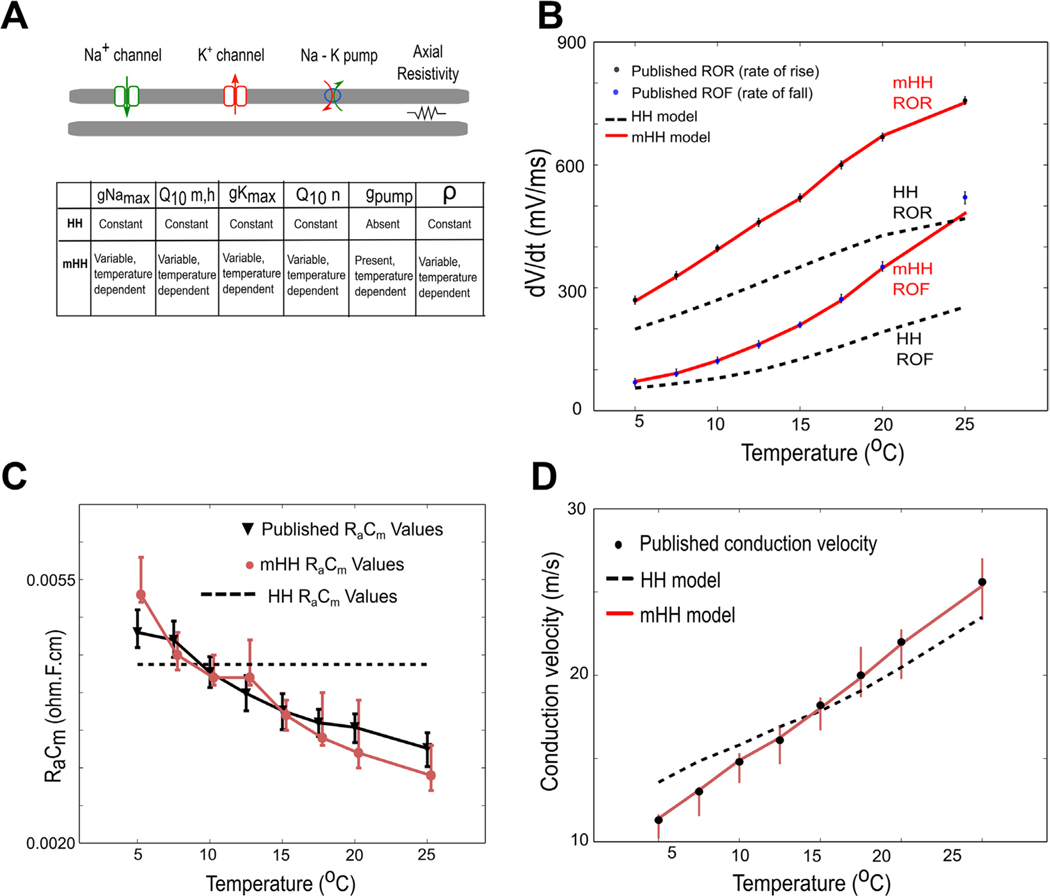
Incorporating temperature dependence into multiple features of the Hodgkin/Huxley model leads to an improved match to experimental data. (A) Schematic indicating how the modified model incorporates more temperature-dependent features than the original Hodgkin/Huxley model. The symbol ρ represents axial resistivity. (B) Comparisons of model outputs to experimental data measuring rates of rise or fall of squid action potentials (dots and error bars correspond to data from Rosenthal and Bezanilla (2000)). Light red line labeled mHH ROR shows the modified Hodgkin/Huxley model fit to data for rate of rise, whereas the black dashed line labeled HH ROR shows the predictions of the unmodified Hodgkin/Huxley model for the rate of rise. Light red line labeled mHH ROF shows the modified Hodgkin/Huxley model fit to data for rate of fall, whereas the black dashed line labeled HH ROF shows the predictions of the unmodified Hodgkin/Huxley model for rate of fall. (C) Comparison of measured product of membrane capacitance and axial resistance ($R_{a}C_{m}$) to values predicted by the two models. Black line, triangles and error bars are values measured by Rosenthal and Bezanilla (2000) in squid giant axons. Light red line, dots and error bars correspond to values predicted by the modified Hodgkin/Huxley model. The dashed line, which is temperature-independent, corresponds to the value used in the original Hodgkin/Huxley model. (D) Comparisons of measured conduction velocity to model predictions. Note that the light red line corresponding to the mHH model yields a better match to the experimental data (dots and error bars) than the black dashed line corresponding to the original Hodgkin/Huxley model.

To capture the response of the neuron to changing temperatures, several features of the original Hodgkin/Huxley model became functions of temperature: (1) a temperature-varying $Q_{10}$ factor for the gating variables of the Hodgkin/Huxley model based on the experimental measurements of Rosenthal and Bezanilla (2000), (2) the peak potassium and sodium conductances varied with temperature, (3) a temperature-dependent sodium/potassium pump, and (4) the axial resistance as a function of temperature (a schematic comparing the original and modified Hodgkin/Huxley model is shown in figure 1(A)). After describing the detailed modifications that were made to the model, the entire set of equations is shown (below, model equations).

#### The ${\text{Q}}_{10}$ factor.

In the standard Hodgkin and Huxley model (Hodgkin and Huxley 1952), temperature dependence is captured by altering the rate constants for the three gates controlling the voltage gated ion channels. The m gate (sodium activation), the h gate (sodium inactivation) and the n gate (potassium activation) are modified to account for changes in temperature. The corresponding modified rate constants are $\alpha_{m}$ and $\beta_{m}$ for the m gate, $\alpha_{h}$ and $\beta_{h}$ for the h gate, and $\alpha_{n}$ and $\beta_{n}$ for the n gate. The values are changed by using the $Q_{10}$ relationship, which is

(4) $$Q_{10}={\left(\frac{R_{2}}{R_{1}}\right)}^{\frac{10}{\left(T_{2}-T_{1}\right)}}$$

where temperature $T_{2}$ is greater than temperature $T_{1}$, rate $R_{2}$ is measured at $T_{2}$, and rate $R_{1}$ is measured at $T_{1}$. If both sides of the equation are raised to the power $\frac{T_{2}-T_{1}}{10}$, it is possible to solve directly for $R_{2}$ at temperature $T_{2}$ as

(5) $$R_{2}=R_{1}Q_{10}^{\frac{T_{2}-T_{1}}{10}}.$$

Thus, for example, to modify the differential equation for the n gate (see below, Model Equations), since rate constants $\alpha_{n}$ and $\beta_{n}$ are multiplied by the same factor to adjust them for temperature, the right-hand side of the entire differential equation for n is multiplied by the $Q_{10}$ term of equation (5). The same factor is used to modify the right-hand sides of the differential equations for the m and h gates. In the original Hodgkin and Huxley model, a fixed value of 3 was used for $Q_{10}$ for all temperature ranges, and the base temperature ($T_{1}$) was set at 6.3 °C (Hodgkin and Huxley 1952). Thus, the factor by which all the differential equations for gates m, n and h were multiplied simplified to $3\frac{T_{2}-6.3}{10}$.

An earlier study of the change in the shape of the action potential (Hodgkin and Katz (1949), their table 2) showed that the $Q_{10}$ for the rates of fall (illustrated in their figure 1(B)), an approximate measure of the activity of the voltage-dependent potassium gates, changed in the temperature ranges 5 °C to 10 °C, 10 °C to 20 °C, and 20 °C to 30 °C. They also observed changes in the $Q_{10}$ for the rate of rise with temperature, though these changes were smaller. These data suggested that the $Q_{10}$ factors multiplying the differential equations for m, n and h were different and varied with temperature. We therefore used a systematic grid search to simultaneously find sets of $Q_{10}$ values for all three gates, varying them from a value of 1.0 (temperature independent) to 3.0 (the original Hodgkin/Huxley model value) in increments of 0.1. We excluded values that did not allow the model to generate an action potential. For those values that did generate an action potential, we determined the total amplitude of the action potential (measured from the resting potential to the peak of the action potential), by comparing these values to the amplitudes actually determined experimentally at that temperature (Hodgkin and Katz (1949), their table 1(c), p 243). Among those sets of $Q_{10}$ values producing total amplitudes within 5% of those measured experimentally, we then evaluated errors for maximum rates of rise and fall of the resulting action potentials (see below, equations (11)–(13)), choosing the $Q_{10}$ values that led to the smallest errors (supplementary figure 1(A) (stacks.iop.org/JNE/16/036020/mmedia)).

As a result of the systematic and exhaustive search, the multiplicative factor was found to be slightly different for each gate, and will be designated as $\phi_{m}(T)$, $\phi_{h}(T)$ and $\phi_{n}(T)$ for the m, h and n gates (respectively). Each of these are piecewise continuous functions:

(6a) $$\phi_{m}(T)=\{\begin{array}{ll} 3^{\frac{T-6.3}{10}}, & 5{}^{\circ}\;\text{C}⩽T⩽10{}^{\circ}\;\text{C},\\ 3^{\frac{10-6.3}{10}}3^{\frac{T-10}{10}}, & 10{}^{\circ}\;\text{C}<T⩽15{}^{\circ}\;\text{C},\\ 3^{\frac{10-6.3}{10}}3^{\frac{15-10}{10}}2.8^{\frac{T-15}{10}}, & 15{}^{\circ}\;\text{C}<T⩽20{}^{\circ}\;\text{C}\\ 3^{\frac{10-6.3}{10}}3^{\frac{15-10}{10}}2.8^{\frac{20-15}{10}}2.7^{\frac{T-20}{10}}, & 20{}^{\circ}\;\text{C}<T⩽25{}^{\circ}\;\text{C} \end{array}$$

(6b) $$\phi_{h}(T)=\{\begin{array}{ll} 3^{\frac{T-6.3}{10}}, & 5{}^{\circ}\;\text{C}⩽T⩽10{}^{\circ}\;\text{C},\\ 3^{\frac{10-6.1}{10}}2.9^{\frac{T-10}{10}}, & 10{}^{\circ}\;\text{C}<T⩽15{}^{\circ}\;\text{C},\\ 3^{\frac{10-63}{10}}2.9^{\frac{15-10}{10}}3^{\frac{T-15}{10}}, & 15{}^{\circ}\;\text{C}<T⩽20{}^{\circ}\;\text{C}\\ 3^{\frac{10-63}{10}}2.9^{\frac{15-10}{10}}3^{\frac{20-15}{10}}3^{\frac{T-20}{10}}, & 20{}^{\circ}\;\text{C}<T⩽25{}^{\circ}\;\text{C} \end{array}$$

(6c) $$\phi_{n}(T)=\{\begin{array}{ll} 3^{\frac{T-6.3}{10}}, & 5{}^{\circ}\;\text{C}⩽T⩽10{}^{\circ}\;\text{C},\\ 3^{\frac{10-63}{10}}2.8^{\frac{T-10}{10}}, & 10{}^{\circ}\;\text{C}<T⩽15{}^{\circ}\;\text{C},\\ 3^{\frac{10-6.3}{10}}2.8^{\frac{15-10}{10}}2.4^{\frac{T-15}{10}}, & 15{}^{\circ}\;\text{C}<T⩽20{}^{\circ}\;\text{C}\\ 3^{\frac{10-63}{10}}2.8^{\frac{15-10}{10}}2.4^{\frac{20-15}{10}}2.3^{\frac{T-20}{10}}, & 20{}^{\circ}\;\text{C}<T⩽25{}^{\circ}\;\text{C}. \end{array}$$

#### Sodium–potassium pump.

In the original Hodgkin and Huxley model, the sodium/potassium pump was not incorporated, because their initial studies suggested that the resting membrane potential was unchanged by temperature (Hodgkin et al 1952). In the studies of Rosenthal and Bezanilla (2000) in *Loglio pealei*, however, the resting potential varied with temperature and season, suggesting that components of the resting potential were temperature dependent. Studies in other molluscan species, such as *Aplysia californica* (Carpenter and Alving 1968) and *Anisodoris nobilis* (Gorman and Marmor 1970), demonstrated that a temperature-dependent sodium–potassium pump significantly contributed to changes in the resting potential with temperature. Rakowski et al (1989) characterized an electrogenic sodium–potassium pump in the squid giant axon that exchanged 3 sodium ions for 2 potassium ions. We used the equations of van Egeraat and Wikswo (1993) for the pump currents, based on the study of Rakowski et al (1989), and incorporated temperature-dependence based on the studies in *Anisodoris* (Gorman and Marmor 1970). We estimated the $Q_{10}$ for the pump conductance from its change in permeability ratio of $P_{\text{Na}}/P_{\text{K}}$ from 0.028 at 4 °C to 0.0068 at 18 °C (Gorman and Marmor 1970), yielding an estimated $Q_{10}$ of 1.88 (using equation (1)), which was then used to scale the peak pump conductance, $g_{\text{NaK}\mathrm{initial}}$. Given that the pump exchanges three sodium ions for two potassium ions, the pump equations are:

(7) $$\begin{array}{c} g_{\text{NaK}}(T)=g_{\text{NaK}\mathrm{initial}}\;1.88^{\frac{\left(T-6.3\right)}{10}},\\ I_{\text{Na\_}\mathrm{pump}}=3\;g_{\text{NaK}}\;(T)\;\left(V_{m}-E_{\text{pump}}\right),\\ I_{\text{K\_}\mathrm{pump}}=-2g_{\text{NaK}}\;(T)\;\left(V_{m}-E_{\text{pump}}\right), \end{array}$$

where $I_{\text{Na\_}\mathrm{pump}}$ and $I_{\text{K\_}\mathrm{pump}}$ are the sodium and potassium currents due to the pump, respectively, $V_{m}$ is the membrane potential, and $E_{\text{pump}}$ is the reversal potential of the pump.

#### Peak sodium and potassium conductances.

In the original Hodgkin and Huxley model, the peak sodium and potassium conductances, ${\bar{g}}_{\text{Na}}$ and ${\bar{g}}_{\text{K}}$, were assumed to be constant and independent of temperature. As reviewed above, more recent work suggests that these values may vary with temperature.

To estimate the peak conductance values, rates of rise and rates of fall were extracted from figure 3 of Rosenthal and Bezanilla (2000) using the program DataThief (www.datathief.org). We extracted the center of the triangle indicating the mean data value as well as the values of the top and bottom error bars to provide a measure of the errors associated with each data point. In the model, the peak of the derivative of the rising phase was used as an estimate of the peak rate of rise, and the peak of the derivative of the falling phase was used as an estimate of the peak rate of fall (supplementary figure 1(B)).

We used a systematic grid search to determine the peak sodium and potassium conductance values that minimized the difference between the rates of rise and fall observed experimentally (Rosenthal and Bezanilla 2000) and those observed in the model (see below for details of how errors were minimized; supplementary figure 1(C)). The data at each of the temperatures reported by Rosenthal and Bezanilla were then fit by exponentials:

(8) $$g_{\text{K}\max}(T)=1.60\;e^{-{\left(\frac{T-27.88}{12.85}\right)}^{2}},\;g_{\text{Na}\max}(T)=0.42\;e^{-{\left(\frac{T-31.83}{31.62}\right)}^{2}}.$$

where the maximum potassium and sodium conductances, $g_{\text{K}\max}$ and $g_{\text{Na}\max}$, have units of S cm^−2^ (supplementary figure 1(D) shows plots of these functions).

#### Axial resistance.

Earlier work showed that the product of the axial resistance and the membrane capacitance could be estimated by the time constant of the rise of the foot of the action potential and its conduction velocity (Jack et al 1975). In our study, the ‘foot’ was defined as the region of the simulated action potential between the time points when the action potential begins (i.e. the time that the potential increased by 1% above the resting membrane potential; another 0.1 ms of data prior to this (100 points) was added to improve the exponential fit) and halfway between this point of onset and the point at which the derivative of the action potential reached its maximum value (supplementary figure 1(B), region indicated by the blue line). The resulting data was fit to an exponential, whose time constant, $\tau_{\text{foot}}$, was then used for estimating the axial resistance (see below). The time constant of the model action potential shown in supplementary figure 1(B) was 179.9 *μ*s.

We calculated conduction velocity as a function of temperature by measuring the time it took the action potential to travel between two points along the axon separated by 8 mm on either side of the midpoint of the axon. For all conduction velocity studies, the model axon was brought to the same uniform temperature along its entire length. The time of arrival of the action potential was determined as the instant when the action potential reached half its maximum value at a specific point on the axon. Conduction velocity was then calculated by dividing the distance between the two points on the axon by the time it took the action potential to travel between the points.

Having measured the time constant, $\tau_{\text{foot}}$, and the conduction velocity, θ, from the model, we then determined the product of the membrane capacitance per unit area ($C_{m}$, in μF cm^−2^) and the axial resistivity ($R_{a},\;\text{kΩ cm}$) from the equation

(9) $$R_{a}C_{m}=\frac{r}{2\tau_{\text{foot}}\theta^{2}}$$

where r is the radius of the axon (Jack et al 1975, pp 116–8). To derive this equation, it is assumed that the conduction velocity of the action potential is large relative to the electrotonic velocity, i.e. the passive propagation of signals along the axon; in addition, it is assumed that the extracellular resistance is negligible relative to the axial resistance.

To illustrate the computation for a specific example: at 5 °C, the time constant of the foot of the simulated action potential, $\tau_{\text{foot}}$, was 179.9 *μ*s (supplementary figure 1(B)); the conduction velocity of that action potential, θ, was 11.3 m s^−1^. The simulated axon diameter is 500 *μ*m, so that its radius is 250 *μ*m. The resultant value of $R_{a}C_{m}$ is $\frac{250\;10^{-6}\;\text{m}}{2\left(179.9\;10^{-6}\;\text{s}\right){\left(11.3\;{\text{m s}}^{-1}\right)}^{2}}$, which equals 0.0054 s m^−1^ or 0.0054 Ω Farads m^−1^. This value is similar to the value shown in figure 5(B) of Rosenthal and Bezanilla (2000) for axons at 5 °C from squid acclimatized to the cooler temperature of May. (Please note that the units shown in their figure 5, ΩFarads cm, do not appear to be correct.) Rosenthal and Bezanilla also determined experimentally that the capacitance of the membrane did not vary significantly from 1 *μ*F cm^−2^ in both warm and cold seasons. Thus, the equation for the axial resistance, $R_{a}(T)$, in Ω cm, as a function of temperature (in °C; the coefficient of determination $R^{2}$ for the fit is 0.94) is

(10) $$R_{a}(T)=56.84\;e^{-0.03\;\text{T}},$$

### Error analysis for average model parameters

To fit parameters to the actual data, we minimized the squared deviations between model measurements for a given set of parameter values and the original data for the rates of rise and fall at each temperature. To capture changes both in the rate of rise and the rate of fall, we used a systematic grid search, varying the peak potassium and sodium conductances from their initial values in the Hodgkin/Huxley model in increments of 0.02 S cm^−2^, and testing for two failure conditions: (1) spontaneous activity in the absence of any excitatory input, which indicated that the potassium conductance was too small to maintain a stable resting potential, and (2) failure to generate an action potential in response to excitatory current, indicating that the potassium conductance was large enough to overwhelm the inward sodium current.

The peak rate of fall was determined in the model action potential by finding the maximum of the derivative of the falling phase (supplementary figure 1(B)). The error in the rate of fall was computed using equation (11):

(11) $$E_{\text{fall}}\;\left(T_{i}\right)=\left|\frac{F_{\text{exp}}\;\left(T_{i}\right)-F_{\text{est}}\;\left(T_{i}\right)}{F_{\text{exp}}\;\left(T_{i}\right)}\right|,$$

where $E_{\text{fall}}\;\left(T_{i}\right)$ is the error at temperature $T_{i}$ during the falling phase of the action potential, $F_{\exp}\;\left(T_{i}\right)$ is the rate of fall measured experimentally at temperature $T_{i}$, and $F_{\text{est}}\;\left(T_{i}\right)$ is the estimated rate of fall from the model at temperature $T_{i}$.

We also computed the error in the experimentally measured rate of rise and that produced by the model. The peak rate of rise was determined from the derivative of the rising phase of the model action potential (supplementary figure 1(B)). Errors in the rate of rise were computed using equation (12):

(12) $$E_{\text{rise}}\;\left(T_{i}\right)=\left|\frac{R_{\text{exp}}\;\left(T_{i}\right)-R_{\text{est}}\;\left(T_{i}\right)}{R_{\text{exp}}\;\left(T_{i}\right)}\right|,$$

where $E_{\text{rise}}\;\left(T_{i}\right)$ is the error at temperature $T_{i}$ in the rising phase, $R_{\text{exp}}\;\left(T_{i}\right)$ is the rate of rise measured experimentally at temperature $T_{i}$, and $R_{\text{est}}\;\left(T_{i}\right)$ is the estimated rate of rise from the model at temperature $T_{i}$.

Total error was then computed by summing the squares of the errors at each temperature over the entire set of temperatures that were matched, which were 5 °C, 7.5 °C, 10 °C, 12.5 °C, 15 °C, 17.5 °C, 20 °C, and 25 °C (based on the measurements of Rosenthal and Bezanilla (2000)). Thus, the final computed error was

(13) $$E_{\text{total}}=\sum_{\text{all}\;T_{i}}{\left(\frac{R_{\exp}\;\left(T_{i}\right)-R_{\text{est}}\;\left(T_{i}\right)}{R_{\text{exp}}\;\left(T_{i}\right)}\right)}^{2}\;\;+\sum_{\text{all}\;T_{i}}{\left(\frac{F_{\text{exp}}\;\left(T_{i}\right)-F_{\text{est}}\;\left(T_{i}\right)}{F_{\text{exp}}\;\left(T_{i}\right)}\right)}^{2}.$$

The total error is plotted on a logarithmic scale (supplementary figure 1(E)) for several variations of the model. The results demonstrate that if only the sodium/potassium pump is temperature dependent (supplementary figure 1(E), second set of bars), or if only the peak sodium and potassium conductances are varied as a function of temperature (supplementary figure 1(E), third set of bars), error is not reduced as much as if both features of the model vary with temperature (supplementary figure 1(E), fourth set of bars). Since Rosenthal and Bezanilla (2002) observed that at 29.5 °C the action potential in *Loligo pealei* was blocked, we used this information to determine the value for $g_{\text{K}\max}$ at this temperature.

Similar optimization was performed for the temperature dependence of the axial resistivity. The simulations were initialized with the standard Hodgkin/Huxley model value of membrane resistivity of 35.4 Ω cm and the conduction velocity at a given temperature $T_{i}$ was evaluated as described above. The resultant conduction velocity was compared with the published results, and membrane resistivity was changed until the modeled conduction velocity was within 1% of the experimentally published values. No other parameters were varied to adjust the conduction velocity.

### Accounting for experimental error

In the original experimental data (Rosenthal and Bezanilla 2000), measurements of the rates of rise and fall had accompanying error bars. We therefore measured not only the mean values shown in the data plots, but the values of the error bars, and propagated those values through the model. This allowed us to provide some estimate of the errors of the parameters, which are the maximum sodium and potassium conductances, the axial resistivity, and the product of $R_{a}C_{m}$. Supplementary figure 1(F) illustrates this for one parameter at one temperature (17.5 °C): the range of measured values for the rates of fall are shown along the x axis; the function relating the rate of fall to the peak potassium conductance is plotted, and the corresponding range in the peak potassium conductance parameter is indicated along the y axis. The arrow points to the measured mean value of the parameter.

### Model equations

These are the model equations that were used in each segment of the NEURON model:

$$\begin{array}{l} C_{m}\;\frac{dV_{m}}{dt}=-g_{L}\left(V_{m}-E_{L}\right)-g_{\text{K}\max}\;(T)\;n^{4}\;\left(V_{m}-E_{\text{K}}\right)\\ \;-g_{\text{Na}\max}\;(T)\;m^{3}h\;\left(V_{m}-E_{\text{Na}}\right)-I_{{\text{Na}}_{\text{pump}}}-I_{{\text{K}}_{\text{pump}}}+I_{\text{segment}}, \end{array}$$

$$\frac{dm}{dt}=\phi_{m}\;(T)\;\left(\alpha_{m}(1-m)-\beta_{m}m\right),$$

$$\frac{dh}{dt}=\phi_{h}\;(T)\;\left(\alpha_{h}(1-h)-\beta_{h}h\right),$$

$$\frac{dn}{dt}=\phi_{n}\;(T)\;\left(\alpha_{n}(1-n)-\beta_{n}n\right),$$

$$\alpha_{n}\;(V)=\frac{-0.01\;(V+55)}{\left(e^{\frac{-(V+55)}{10}}-1\right)}$$

$$\beta_{n}\;(V)=0.125\;\left(e^{\frac{-(V+65)}{80}}\right)$$

$$\alpha_{m}\;(V)=\frac{-0.1\;(V+40)}{\left(e^{\frac{-(V+40)}{10}}-1\right)}$$

$$\beta_{m}\;(V)=4\;\left(e^{\frac{-(V+65)}{18}}\right)$$

$$\alpha_{h}\;(V)=0.07\;\left(e^{\frac{-(V+65)}{20}}\right)$$

$$\beta_{m}\;(V)=\frac{1}{\left(e^{\frac{-(V+35)}{10}}+1\right)}$$

$$g_{\text{K}\max}(T)=1.60\;e^{-{\left(\frac{T-27.88}{12.85}\right)}^{2}},$$

$$g_{\text{Na}\max}(T)=0.42\;e^{-{\left(\frac{T-31.83}{31.62}\right)}^{2}}$$

$$g_{\text{NaK}}\;(T)=g_{\text{NaK}\mathrm{initial}}\;2^{\frac{\left(T-6.3\right)}{10}}$$

$$I_{\text{Na}\mathrm{pump}}=3g_{\text{NaK}}(T)\;\left(V_{m}-E_{\text{pump}}\right)$$

$$I_{\text{K}\mathrm{pump}}=-2g_{\text{NaK}}(T)\;\left(V_{m}-E_{\text{pump}}\right)$$

$$I_{\text{segment}}=\frac{V_{j-1}-V_{j}}{r_{a,j-1,j}}-\frac{V_{j}-V_{j+1}}{r_{a,j,j+1}}$$

$$\phi_{m}\;(T)=\{\begin{array}{ll} 3^{\frac{T-6.3}{10}}, & 5{}^{\circ}\;\text{C}⩽T⩽10{}^{\circ}\;\text{C},\\ 3^{\frac{10-6.3}{10}}3^{\frac{T-10}{10}}, & 10{}^{\circ}\;\text{C}<T⩽15{}^{\circ}\;\text{C},\\ 3^{\frac{10-6.3}{10}}3^{\frac{15-10}{10}}2.8^{\frac{T-15}{10}}, & 15{}^{\circ}\;\text{C}<T⩽20{}^{\circ}\;\text{C}\\ 3^{\frac{10-6.3}{10}}3^{\frac{15-10}{10}}2.8^{\frac{20-15}{10}}2.7^{\frac{T-20}{10}}, & 20{}^{\circ}\;\text{C}<T⩽25{}^{\circ}\;\text{C} \end{array}$$

$$\phi_{m}\;(T)=\{\begin{array}{ll} 3^{\frac{T-6.3}{10}}, & 5{}^{\circ}\;\text{C}⩽T⩽10{}^{\circ}\;\text{C},\\ 3^{\frac{10-6.3}{10}}2.9^{\frac{T-10}{10}}, & 10{}^{\circ}\;\text{C}<T⩽15{}^{\circ}\;\text{C},\\ 3^{\frac{10-6.3}{10}}2.9^{\frac{15-10}{10}}3^{\frac{T-15}{10}}, & 15{}^{\circ}\;\text{C}<T⩽20{}^{\circ}\;\text{C}\\ 3^{\frac{10-6.3}{10}}2.9^{\frac{15-10}{10}}3^{\frac{20-15}{10}}3^{\frac{T-20}{10}}, & 20{}^{\circ}\;\text{C}<T⩽25{}^{\circ}\;\text{C} \end{array}$$

$$\phi_{n}\;(T)=\{\begin{array}{ll} 3^{\frac{T-6.3}{10}}, & 5{}^{\circ}\;\text{C}⩽T⩽10{}^{\circ}\;\text{C},\\ 3^{\frac{10-6.3}{10}}2.8^{\frac{T-10}{10}}, & 10{}^{\circ}\;\text{C}<T⩽15{}^{\circ}\;\text{C},\\ 3^{\frac{10-6.3}{10}}2.8^{\frac{15-10}{10}}2.4^{\frac{T-15}{10}}, & 15{}^{\circ}\;\text{C}<T⩽20{}^{\circ}\;\text{C}\\ 3^{\frac{10-6.3}{10}}2.8^{\frac{15-10}{10}}2.4^{\frac{20-15}{10}}2.3^{\frac{T-20}{10}}, & 20{}^{\circ}\;\text{C}<T⩽25{}^{\circ}\;\text{C} \end{array}$$

$$R_{a}\;(T)=56.84\;e^{-0.03\;\text{T}}$$

$$r_{a}(T)=\frac{R_{a}\;(T)}{\pi r^{2}/4}.$$

### Fixed parameter values

**Table:** 

Parameter	Symbol	Value (units)
Leak conductance	$g_{L}$	0.3 mS cm^−2^
Membrane capacitance	$C_{m}$	1 *μ*F cm^−2^
Maximum pump conductance	$g_{\text{NaK}}$	7 *μ*S cm^−2^
Pump reversal potential	$E_{\text{pump}}$	−220 mV
Squid giant axon radius	r	0.25 mm
Leak reversal potential	$E_{L}$	−51 mV
Potassium ion Nernst potential	$E_{\text{K}}$	−74 mV
Sodium ion Nernst potential	$E_{\text{Na}}$	53 mV

### Code availability

The simulation environment NEURON was scripted using Python to search for parameter values while minimizing errors, as described above. The Python package ‘numpy’ was used for numerical analysis, and the package ‘matplotlib’ was used for plotting data. Fits of data to curves were done using MATLAB’s Curve Fitting toolbox. All code used in the modeling and optimization for this paper are available for download under the appropriate licenses from the GitHub repository: (https://github.com/mohitganguly/IRBlock_Vanderbilt).

#### Compensatory current

To test the role of voltage-dependent ion channels on thermal block, the conductances of all or some of the channels were set to zero, similar to experimental manipulations such as applying the drug TTX to block voltage-dependent sodium ion channels, or the drug TEA to block voltage-dependent potassium ion channels. Doing so has a significant side effect: the resting membrane potential changes, as it depends in part on the currents through the voltage-dependent ion channels. Thus, to separate the effects of removing ion channels from the resulting changes in resting membrane potential, we used compensatory current, which is based on the concept of dynamic clamp (Prinz et al 2004) to restore the membrane potential to its original resting potential. Conceptually, the idea is simple: one can computationally ‘remove’ a particular ionic current by computing the value of current that should be generated through that ion channel, and then inject the opposite amount of current; a similar approach can be used to computationally ‘add’ a particular ionic current.

To show a specific example of how this was done, in supplementary figure 2(A), the schematic lower axon has a central section whose peak potassium conductance, $g_{\text{K}\max}$, is set to zero (indicated by the orange rectangle). The schematic lower axon has its potassium channel conductances set to zero in the central region (indicated by the red rectangle). Into each segment of the central region in the lower axon, a compensatory current clamp is inserted (using a new NEURON function, specifically written for this purpose, kd.mod). For each of the segments in this central section, corresponding to the identical segment in the axon above it, the program determines the current of the upper axon, $I_{\text{K}}(x,t)$, at that location over time, and injects the negative of this current into that segment of the lower axon, $I_{\text{clamp}}$. The injected current negates the current change due to the absent ion channels in the lower axon. Thus, the compensatory current clamp restores the lower axon to its original resting potential despite the absence of the potassium ion channels.

To provide an objective measure of the discrepancy between the original values and those in the axon receiving compensatory current, we computed the mean square error between the peak voltage changes along the control axon as compared to the peak voltage changes along the axon whose central region was subjected to compensatory current. Supplementary figure 2(B) is a plot of the mean square error as the step size for the compensatory current is varied, demonstrating the importance of using a very small step size to keep the error low because of the high conduction velocity of the squid giant axon.

#### Incorporating temperature dependence improves model accuracy

Incorporating temperature-dependence into the peak sodium and potassium conductances, the sodium/potassium pump, and the axial resistance led to model outputs that more closely matched experimental observations in squid giant axons (Rosenthal and Bezanilla 2000) than those predicted by the original Hodgkin/Huxley model. Modified model predictions of the influence of temperature on rates of action potential rise and fall (figure 1(B)), axial resistance multiplied by membrane capacitance (figure 1(C)), and conduction velocity (figure 1(D)) better matched the experimental results than those predicted by the original Hodgkin/Huxley model.

## Results

### Increasing temperature leads to a net increase in hyperpolarizing current

**Figure 2.**
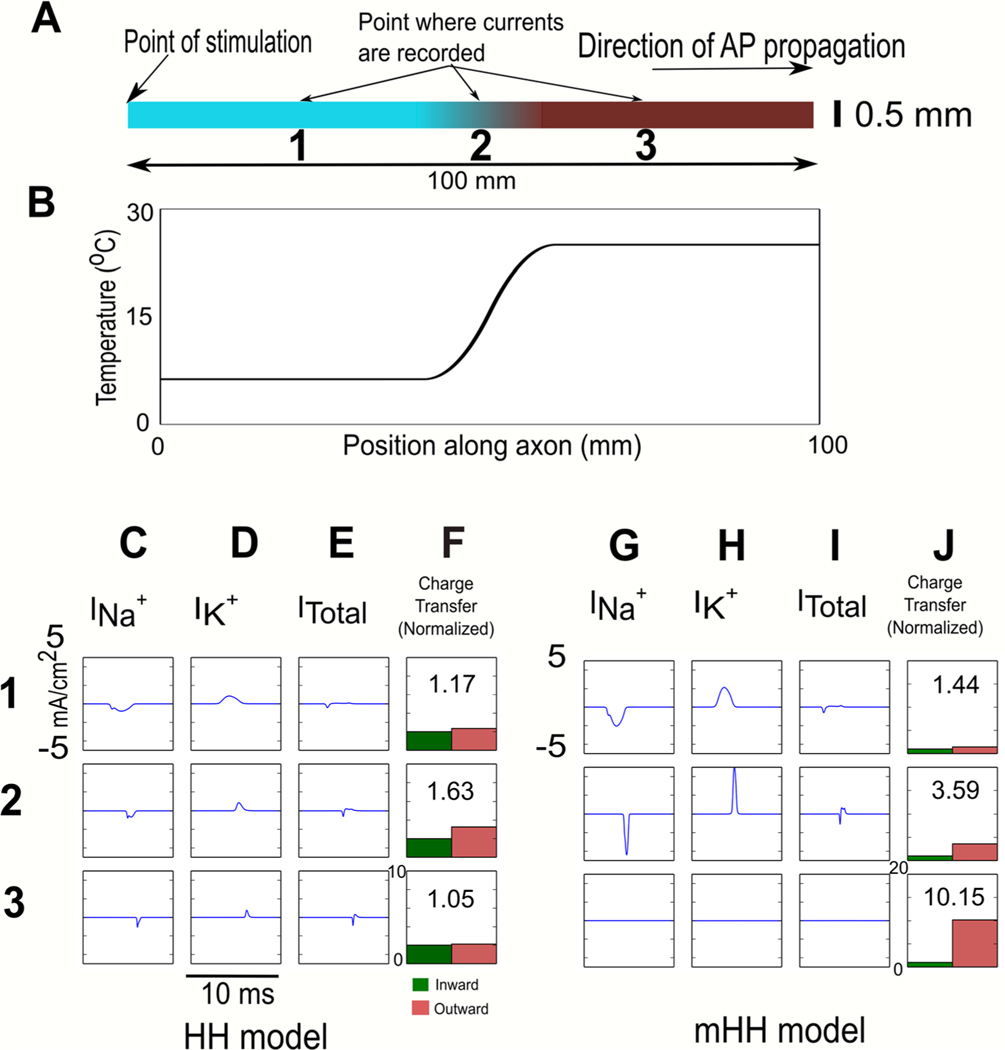
Net increase in hyperpolarizing current in the central region of an axon in response to an action potential as the axon’s temperature is gradually increased from 6.3 °C to 25 °C around the center of the axon. (A). Schematic of experiment. The left-hand side of the model axon (0.5 mm diameter) was held at 6.3 °C, and then, from 40 to 60 mm from its left-hand side, its temperature was gradually increased to 25 °C (see methods, simulating temperature changes in the model, equations (2) and (3)). An action potential was initiated using a current pulse of 2000 nA (1 ms duration) injected into the left-hand side of the axon, and the sodium, potassium and net currents were measured at the center of the axon. Sodium current, potassium current, net current and charge transfer were measured at three positions: position 1—region of the axon at the control temperature (25% along its length). Position 2—center of axon, region within which temperature is changing (50% along its length). Position 3—region of the axon at the new, higher temperature (75% along its length). (B) Temperature profile along the axon, ranging from 6.3 °C to 25 °C from 40 mm to 60 mm. (C)–(F) Results for the Hodgkin/Huxley model. (C) Inward sodium current. (D) Outward potassium current. (E) Net current. (F) Inward versus outward charge transfer. (G)–(J) Results for the modified Hodgkin/Huxley model. (G) Inward sodium current. (H) Outward potassium current. (I) Net current. (J) Inward versus outward charge transfer. Both models show a net increase in hyperpolarizing (outward) current in the central region as temperature increases in response to an action potential; in the modified model, the net outward current is also larger in the region of elevated temperature, although the currents are much smaller.

We first determined whether adding temperature-dependence to components of the original Hodgkin/Huxley led to qualitatively new behavior, or primarily altered the quantitative response of the modified model to temperature. Since most temperature manipulations generate a smooth change from an initial temperature to a final temperature, we generated an action potential along a model axon whose first half was at the control temperature of 6.3 °C, and then smoothly changed the temperature of the second half of the model axon to 25 °C (see equations (2) and (3), Methods; figures 2(A) and (B)). By generating the temperature change near the middle of the axon, we ensured that initial currents at the point of action potential initiation were gone by the time the action potential encounter ed the change in temperature. The consequence of a gradual change in temperature was a clear increase in net hyperpolarizing (outward) current for both the original Hodgkin/Huxley model (figures 2(C)–(F), position 2) and for the modified model (figures 2(G)–(J), position 2) at the center of the region undergoing a change in temperature. In the region at the control temperature, the relative ratio of outward to inward current was more similar for both models (figures 2(C)–(F) and (G)–(J), position 1). In the region that was at the new, higher temperature in the modified model, the ratio of outward to inward current was also increased (figures 2(G)–(J), position 3), although the currents were much smaller.

**Figure 3.**
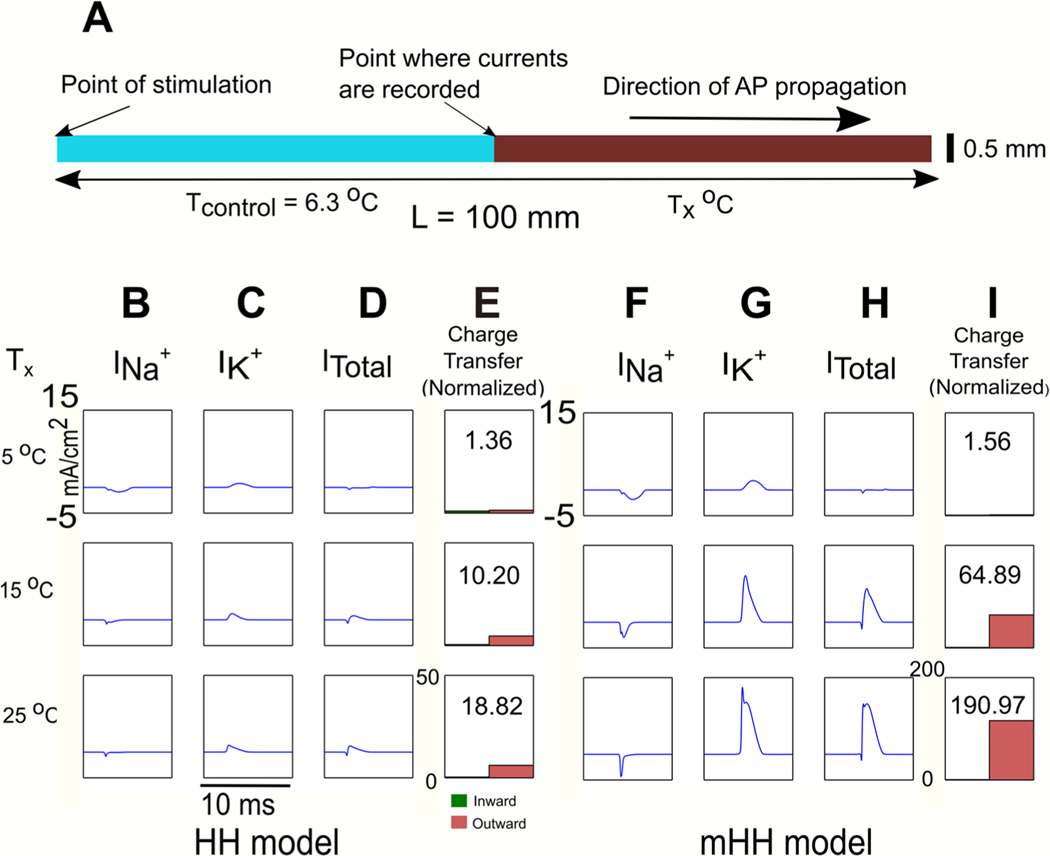
Sharply increasing temperature at the center of an axon leads to a net increase in hyperpolarizing current in response to an action potential. (A) Schematic showing that the left half of the model axon (0.5 mm diameter) was kept at the control temperature of 6.3 °C, whereas the right-hand side was kept at temperatures of 5 °C, 15 °C or 25 °C. An action potential was initiated using a current pulse (2000 nA, 1 ms duration), and currents were measured at the middle of the axon, right at the transition between the control and the experimental temperatures. (B)–(E) Measurements of sodium current ($I_{\text{Na}}$), potassium current ($I_{\text{K}}$), total current ($I_{\text{total}}$), and normalized charge transfer at 5 °C, 15 °C and 25 °C for the Hodgkin/Huxley model (respectively). Area under curves in parts (B) and (C) were computed (total sodium or potassium currents, respectively), and then normalized to the inward sodium current predicted by the Hodgkin/Huxley model at 5 °C (currents are measured in units of mA cm^−2^; when integrated over time, the resulting units are pC cm^−2^). The actual sodium current at 5 °C used for normalization was 0.27 pC cm^−2^. (F)–(I) Results of the same measurements for the modified Hodgkin/Huxley model. Note that for both models, the ratio of outward to inward current (number at the top of the box in sections (E) and (I)) increases as temperature increases. Thus, with increasing temperature, a net hyperpolarizing current develops in response to the action potential.

To better understand the development of the net hyperpolarizing current as a function of temperature, we confined the temperature change to a single step change (figure 3), since in figure 2, we observed that the changes in currents primarily occur where the axon shows significant changes in temperature. Both the original Hodgkin/Huxley model and the modified model qualitatively showed similar responses: as temperature increased from 5 °C to 25 °C, the sodium inward current became shorter (figures 3(B) and (F), for Hodgkin/Huxley model and for modified model, respectively), due to the increased speed of sodium ion channel inactivation. Over the same temperature range, the potassium outward current became faster and larger (figures 3(C) and (G), for Hodgkin/Huxley model and modified model, respectively), due to the increased speed of potassium ion channel activation. As a consequence, the net current ($I_{\text{total}}$) became increasingly outward (hyperpolarizing) with increasing temperature as the membrane was depolarized by the action potential (figures 3(D) and (H) for Hodgkin/Huxley model and modified model, respectively). For both models, much more outward charge was transferred relative to inward charge (figures 3(E) and (I)).

### Voltage-dependent ion channels are necessary for thermal block

**Figure 4.**
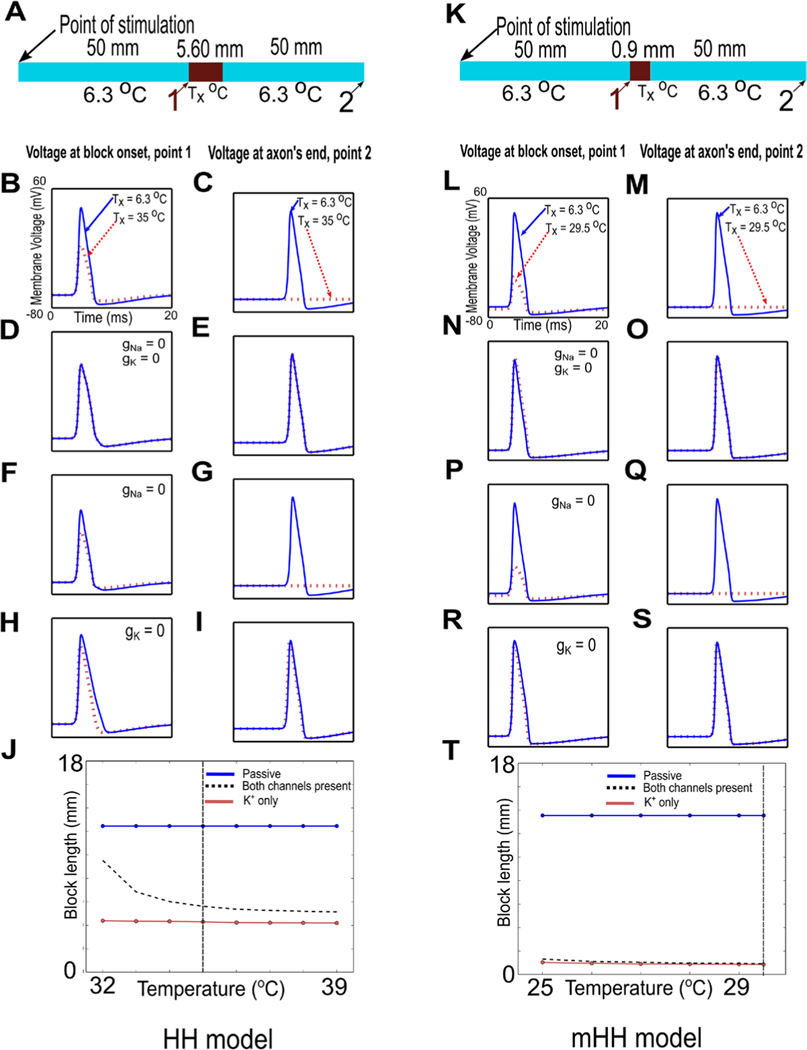
Voltage-gated ion channels are critical for thermal block, and voltage-dependent potassium channels dominate the block at higher temperatures. (A) and (K) Schematic of the experiment. The central region of a model axon was changed to a temperature, $T_{x}$, that blocked action potential propagation for the original Hodgkin/Huxley model (A) and for the modified model (K). Regions of the axon before and after the central region were kept at the control temperature of 6.3 °C. An action potential was initiated at the left-hand side of the model axon with a current of 2000 nA (1 ms duration), and the propagating action potential was measured as it reached the region of changed temperature (point labeled 1) and at the end of the axon (point labeled 2). (B), (C), (L) and (M) Control responses. When the central region remained at 6.3 °C, the action potential propagated normally through the region and reached the end of the axon ((B), (C), (L) and (M), solid blue lines). As the action potential reached the central region subjected to a higher temperature, it was reduced in amplitude ((B) and (L), dashed red lines; compare to solid blue lines), and it was completely blocked at the end of the axon ((C) and (M), dashed red lines). (D), (E), (N) and (O) The conductances of both the voltage-gated sodium ion and potassium ion channels were set to zero in the central region. When the temperature of the central region was not changed, the action potential shape changed slightly as it reached the region without ion channels ((D) and (N), solid blue lines), but by the end of the axon, its shape was again similar to the control action potential ((E) and (O), solid blue lines). When the central region is subjected to an increase in temperature, there is no change in the action potential in the Hodgkin/Huxley model ((D) and (E); the dashed red lines are completely covered by the solid blue lines). A slight widening of the action potential is visible in the modified Hodgkin/Huxley model as the action potential reaches the region at a higher temperature ((N), dashed red line), but the action potential at the end of the axon is identical to that generated when the central region is at its original temperature ((O); dashed red line is covered by solid blue line). (F), (G), (P) and (Q) Voltage-dependent potassium channels are sufficient to induce thermal block. The conductance of the voltage-dependent sodium ion channels was set to zero in the central region. The peaks of the action potentials were slightly reduced when the central region was at its original temperature ((F) and (P), solid blue lines), but the action potentials at the end of the axon were similar to control action potentials ((G) and (Q), solid blue lines). In contrast, increasing the temperature of the central region blocked action potentials. The action potential was reduced as it reached the region of increased temperature ((F) and (P), dashed red lines), and was completely absent at the end of the axon ((G) and (Q), dashed red lines). (H), (I), (R) and (S) Voltage-dependent potassium channels are necessary to induce thermal block. The conductance of the voltage-dependent potassium ion channels was set to zero in the central region. As the action potentials reached the central region, they were slightly wider than normal ((H) and (R), solid blue lines), but were similar to control action potentials at the end of the axon ((I) and (S), solid blue lines). Action potentials were not blocked by increased temperature; they narrowed as they reached the region of increased temperature ((H) and (R), dashed red lines), but were essentially similar to control action potentials at the end of the axon ((I) and (S), dashed red lines; in (S), the solid blue line covers the dashed red line). (J) and (T) Minimum lengths required to block the action potential when all voltage-gated ion channels are intact (dashed red line) are similar to those needed to block when only voltage-gated potassium ion channels are present (light red line), especially at higher temperatures. At low temperatures, the original Hodgkin/Huxley model is also affected by the effects of temperature on the voltage-gated sodium ion channels. At high temperatures, the voltage-gated potassium ion channels clearly dominate the block length (J), and these channels dominate at all temperatures in the modified model (T). The dashed vertical lines in (J) and (T) (at 35 °C and 29.5 °C, respectively), correspond to the results shown in parts (A)–(I) and (K)–(S) of this figure, respectively.

In both models, increased temperature alters both the sodium and the potassium ion channels, so both could contribute to thermal block. To determine the contributions of the voltage-dependent ion channels to thermal block, we changed the temperature of a model neuron in its center while selectively removing ion channels computationally from that central region, and then initiating an action potential at one end of the axon. We recorded the voltage changes at the beginning of the central region at which the temperature change occurred, and at the end of the model axon (figures 4(A) and (K), points marked 1 and 2). In both models, when all ion channels were present, we determined a minimum length and temperature at which action potential propagation was blocked (35 °C applied to a 5.6 mm length at the center of the axon for the Hodgkin/Huxley model, 29.5 °C applied to an 0.9 mm length at the center of the axon for the modified model).

As the action potential reached the region of changed temperature, its amplitude was significantly reduced (solid red lines in figures 4(B) and (L) for the Hodgkin/Huxley and modified Hodgkin/Huxley models, respectively; dashed blue lines show the action potential when the central region is at the control temperature of 6.3 °C). No action potential was recorded at the end of the model axon (solid red lines in figures 4(C) and (M) for the Hodgkin/Huxley and modified Hodgkin/Huxley models, respectively; dashed blue lines show the action potential when the central region is at the control temperature—the action potential propagates through the central region unchanged to the end of the axon).

To demonstrate that all of the voltage-gated ion channels were necessary for thermal block, we set the conductances of both the sodium and potassium voltage-gated ion channels to zero within the central region. Removing ion channels can change the resting potential of the central region because the voltage-dependent ion channels contribute significantly to the resting potential. Since, at rest, the membrane is primarily permeable to potassium ions, removing all ion channels will tend to depolarize the membrane. In turn, a depolarized membrane will alter the states of voltage-dependent ion channels on either side of the central region. To eliminate this potentially confounding factor, we applied compensatory current to the central region to ensure that it remained at the control resting potential after ion channels were removed computationally (see methods, compensatory current, and supplementary figure 2).

When the action potential encountered the central region, its amplitude decreased and the waveform broadened (figures 4(D) and (N), dashed blue lines). By the time the action potential reached the end of the model axon, it was identical to the control action potential (figures 4(E) and (O), dashed blue lines). Removing all the voltage-dependent ion channels did not prevent passive currents from propagating through the region in which the ion channels were blocked, and these passive currents re-activated an action potential that propagated to the end of the model axon.

When the central region was subjected to an increased temperature, the Hodgkin/Huxley model showed no changes (figures 4(D) and (E); the solid red lines corresponding to the increased temperature condition are completely covered by the dashed blue lines). The lack of response is predictable, as all of the temperature-dependent aspects of the model have been removed. In the modified Hodgkin/Huxley model, both the axial resistance (figure 1(D)) and the sodium/potassium pump depend on temperature. When the central region was subjected to an increased temperature, the action potential showed a small increase in width relative to the control (figure 4(N), dashed red line), but the action potential at the end of the axon was essentially unchanged (figure 4(O); the dashed red line is covered by the control solid blue line). Thus, both models clearly require the voltage-dependent ion channels to generate thermal block in response to increased temperature.

### At higher temperatures, voltage-gated potassium channels are necessary and sufficient for thermal block

To determine if voltage-gated sodium ion channels are necessary for thermal block, we set the conductance of the voltage-gated sodium ion channels to zero. We did not modify the conductance of the voltage-gated potassium ion channels. Again, compensatory current was used to ensure that the resting potential was unchanged by computationally removing the voltage-gated sodium ion channels. In both models, when the central region was kept at the control temperature of 6.3 °C, the action potential peak was reduced as it reached the central region (figures 4(F) and (P) for the Hodgkin/Huxley and modified models, respectively, solid blue lines). Passive currents were able to propagate through the region and re-activate an action potential, which arrived at the end of the axon essentially unchanged (figures 4(G) and (Q), respectively, solid blue lines). When the central region was subjected to increased temperature, the peak of the action potential was significantly reduced in both models as it reached the region of increased temperature (figures 4(F) and (P), respectively, dashed red lines). The action potential was completely blocked by the end of the axon (figures 4(G) and (Q), respectively, dashed red lines). These results demonstrate that voltage-gated sodium ion channels are not necessary, whereas voltage-gated potassium ion channels are alone sufficient to induce thermal block.

To determine if voltage-gated potassium channels are necessary for thermal block, we set the conductance of these channels to zero, but did not modify the conductance of the voltage-gated sodium ion channels. Again, we used compensatory current to ensure that the resting potential was unchanged despite the computational removal of the voltage-gated potassium ion channels. In both models, when the central region was kept at the control temperature of 6.3 °C, the action potential broadened slightly as it reached the central region (figures 4(H) and (R) for the Hodgkin/Huxley and modified models, respectively, solid blue lines), but propagated normally to the end of the axon (figures 4(I) and (S), respectively, solid blue lines). When the central region was subjected to increased temperature, the action potential narrowed slightly as it reached the central region (figures 4(H) and (R), respectively, dashed red lines), but propagated through and reached the end of the axon essentially unchanged (figures 4(I) and (S), respectively, dashed red lines). Under these conditions, voltage-gated potassium ion channels are necessary for thermal block, and voltage-gated sodium channels are not sufficient alone to induce thermal block.

To generalize these results, minimum block lengths for all four conditions (all voltage-gated ion channels intact, all voltage-gated ion channels blocked, voltage-gated sodium ion channels blocked, or voltage-gated potassium ion channels blocked) were measured in both models over a wide range of temperatures, with compensatory current applied to ensure no change from the resting potential after computational removal of ion channels. Since voltage-gated sodium ion channels contribute depolarizing current and voltage-gated potassium ion channels contribute hyperpolarizing current, the block lengths when all ion channels are intact should lie midway between the block lengths for either of the two voltage-gated ion channels alone. If one of the voltage-gated ion channels dominates, then the block lengths when all ion channels are intact should lie closer to the block lengths for that ion channel alone. The data suggest that, at the lowest temperature that can cause block in the Hodgkin/Huxley model, both voltage-gated sodium and potassium ion channels contribute to the block, but as the temperature is raised, the block length is dominated by the voltage-gated potassium ion channels (figure 4(J)). In contrast, for the modified Hodgkin/Huxley model, at all temperatures capable of inducing block, the block length associated with the potassium channels dominates (figure 4(T)).

As another way of testing the role of the different voltage-dependent ion channels in thermal inhibition, we changed the temperature-dependence of the gating variables m, h and n in the modified Hodgkin Huxley model. By setting the $Q_{10}$ of n to 1, we ensured that the voltage-dependent potassium ion channels would not open more rapidly as the axon temperature increased. After this modification, application of increased temperature did not induce block of the action potential (data not shown). In contrast, after setting the $Q_{10}$ of m and h to 1, eliminating the temperature-dependence of the voltage-dependent sodium ion channels, increased temperature could still induce block (data not shown). Because it is not currently feasible to experimentally eliminate the temperature-dependence of ion channels, we did not pursue this approach further.

Applying compensatory current to an axon may be experimentally challenging, so we determined whether qualitatively similar results were observed if the central region’s resting potential was allowed to change after computationally removing voltage-dependent ion channels in either the original Hodgkin/Huxley model or the modified model (supplementary figures 3(A) and (F)). As expected, in both models, computational removal of all voltage-dependent ion channels from the central region caused a small depolarization of the central region and regions around it (supplementary figures 3(B) and (G)); computational removal of all voltage-dependent potassium ion channels caused a larger depolarization (supplementary figures 3(C) and (H)); and computational removal of all voltage-dependent sodium ion channels caused a small hyperpolarization (supplementary figures 3(D) and (I)). Despite these changes in potential, the qualitative results we obtained for length of block needed as a function of temperature were very similar to those obtained using compensatory current (supplementary figures 3(E) and (J); compare figures 4(J) and (T)): at higher block temperatures, the length of the block was clearly dominated by the voltage-dependent potassium ion channels. In the Hodgkin/Huxley model, at lower block temperatures, the voltage-dependent sodium ion channels clearly played an important role in determining block length (supplementary figure 3(E)). In the absence of compensatory current, the central region of the axon depolarizes once the voltage-gated potassium ion channels are blocked. In turn, this depolarization reduces the inward current through the voltage-dependent sodium ion channels because they inactivate in response to depolarization. The depolarizing current is also partially shunted by the increased conductance when the voltage-dependent sodium ion channels are activated. The net result is the same: the block lengths when all ion channels are intact lie between block lengths for either of the two voltage-gated ion channels alone.

**Figure 5.**
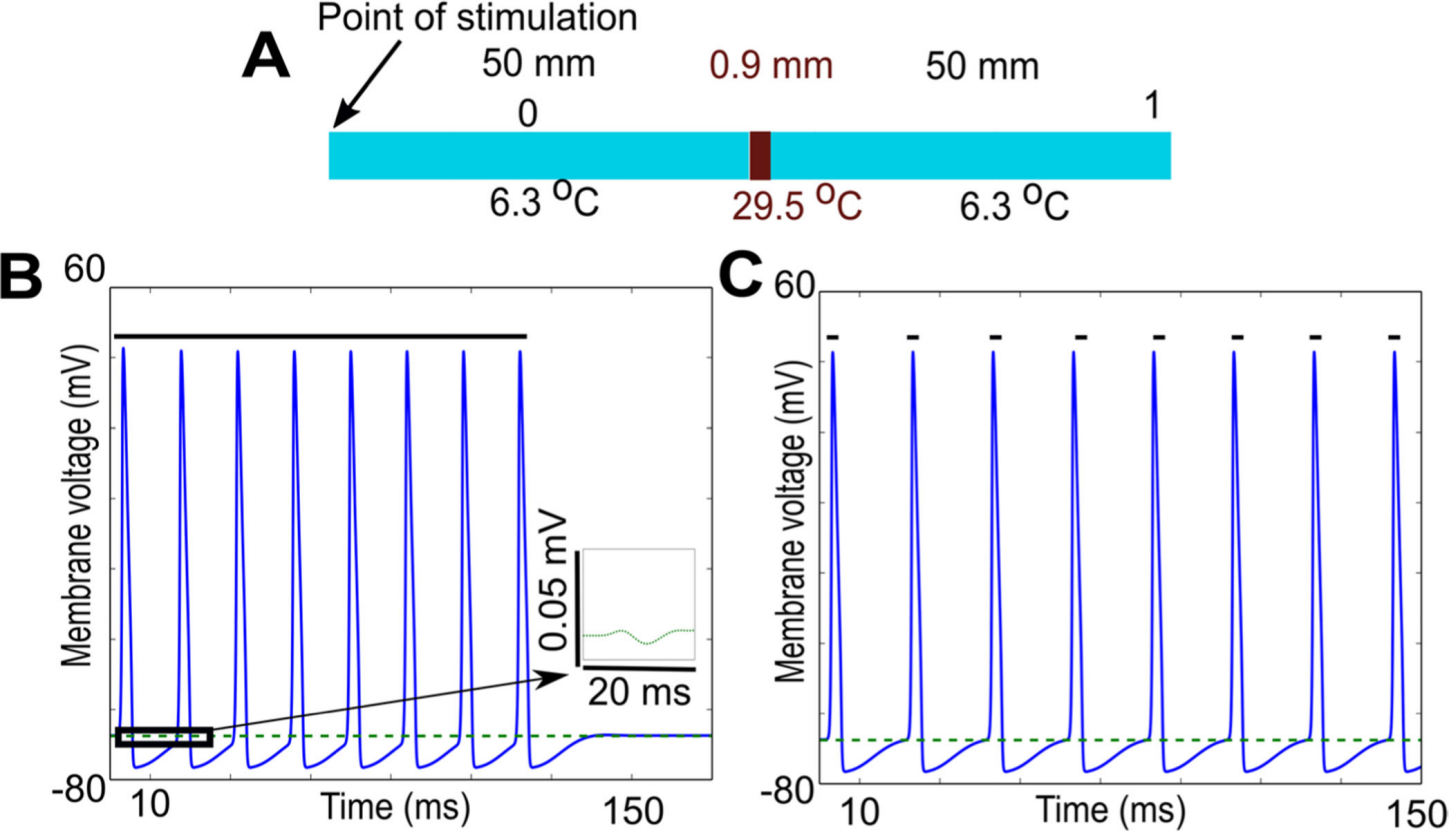
Block of single action potentials also blocks repetitive firing. (A) Schematic of experiment: current was injected on the left-hand side, the axon was raised to a temperature sufficient to induce block of a single propagating action potential, and potentials were recorded at the points labeled 0 and 1. (B) Repetitive firing (80 Hz) due to a steady depolarizing pulse (2000 nA; duration of the current is indicated by the dark green line above the action potentials) is completely blocked by the same conditions that block a single action potential. Action potentials are measured prior to the block region (black line, at 25% of the length of the axon; point labeled 0 on the schematic diagram), and membrane potential is measured at the end of the axon (dashed green line, at 90% of the length of the axon, point labeled 1 on the schematic diagram). Small residual filtered versions of the original action potential appear at the end of the axon, but the action potential does not propagate past the block region (see inset, which shows an expanded view of the dashed green line contained within the box; the potential measured at the end of the axon is a small passive response). (C) Repetitive firing (53.33 Hz) due to individual current pulses (2000 nA, 1 ms duration, 15 ms period; small dark green lines above each action potential indicate time of current injection) is completely blocked by the same conditions that block a single action potential. Once again, the action potentials propagate normally until the block region (black line, measured at the point labeled 0 in the schematic), and only small residual passive versions appear at the end of the axon (dashed green line, measured at the point labeled 1 in the schematic), but the action potential does not propagate past the block region.

Since neurons generally fire repetitively in response to stimuli, we tested whether a thermal block that was sufficient to stop an action potential could also block repetitive firing (figure 5(A)). Thermal block sufficient to block a single action potential was able to block repetitive firing, whether induced by a steady depolarizing current (figure 5(B)), or by individual depolarizing current pulses (figure 5(C)).

### Thermal block length scales with the square root of axon diameter

Unmyelinated fibers range widely in size across phylogeny; some are as small as 0.2 *μ*m (e.g. unmyelinated C fibers in a muscle nerve or a cutaneous nerve; Gardner and Johnson 2013). We sought to determine the minimal thermal block lengths as a function of fiber diameter. A mathematical analysis of the cable equation predicted that modalities acting on the surface of axons would scale with the square root of the diameter of the axon (Lothet et al 2017, supplemental material). In the mathematical model, a generalized form of the cable equation was derived in which the dependence of membrane capacitance and resistance and axial resistance on axon diameter was made explicit. The equation was then rewritten to re-scale length by the square root of the axon diameter, and in this new coordinate system, the dependence of all the terms on axon diameter could be factored out, making the new equation independent of axon diameter. In turn, this immediately implied that if one axon has diameter $d_{1}$, and could be blocked by a high-temperature region whose minimum length was L, then an axon of diameter $d_{2}$ would be blocked by a high temperature region of minimum length $L\sqrt{\frac{d_{2}}{d_{1}}}$. These mathematical predictions have been confirmed by the experimental studies in Lothet et al (2017), which demonstrated that thermal inhibition could selectively block small-diameter axons before large-diameter axons in both an invertebrate nerve (from *Aplysia californica*) and the vertebrate vagus (from the musk shrew *Suncus murinus*). The conduction velocity and length constant of an unmyelinated axon also scale with the square root of axon diameter (Hodgkin 1954, Debanne et al 2011). These observations suggest that the spread of current away from the region of increased temperature should also scale as the square root of the axon diameter. Thus, if voltage changes were plotted against the distance scaled by the length constant of the axon, which is proportional to the square root of the axon diameter, the resulting voltage changes should be independent of axon diameter.

The length constant of an unmyelinated axon is defined from the solution of the cable equation after time-dependent changes in voltage have died out while a steady current is injected into the cable, yielding

(14) $$V_{m}\;(x)=V_{0}e^{-x/\lambda },$$

where $V_{m}(x)$ is the membrane potential $V_{m}$ at point x along the cable, $V_{0}$ is the voltage at the point of current injection, and λ is the length constant (Jack et al 1975). Note that when the original voltage $V_{0}$ has fallen by 1/e of its original value, x=λ. Thus, to determine the length constant of an axon with a region of increased temperature, we injected a steady hyperpolarizing current, and determined the distance at which the hyperpolarization had fallen to a value of 1/e of its value at the point of injection. For a 500 *μ*m axon, this value was 3.96 mm. The length constant of the same axon at its original temperature was 3.93 mm (a difference of 1%). To determine how the current spread scaled with axon diameter, an action potential and its blocked version were also determined for a 10 *μ*m diameter axon, and then rescaled by the length constant (the length constant was 0.56 mm; note that 3.96 is equal to 0.56 times the square root of the ratio of the diameters, i.e. $0.56\times \sqrt{\frac{500}{10}}=3.96$, as predicted by the square root scaling law).

**Figure 6.**
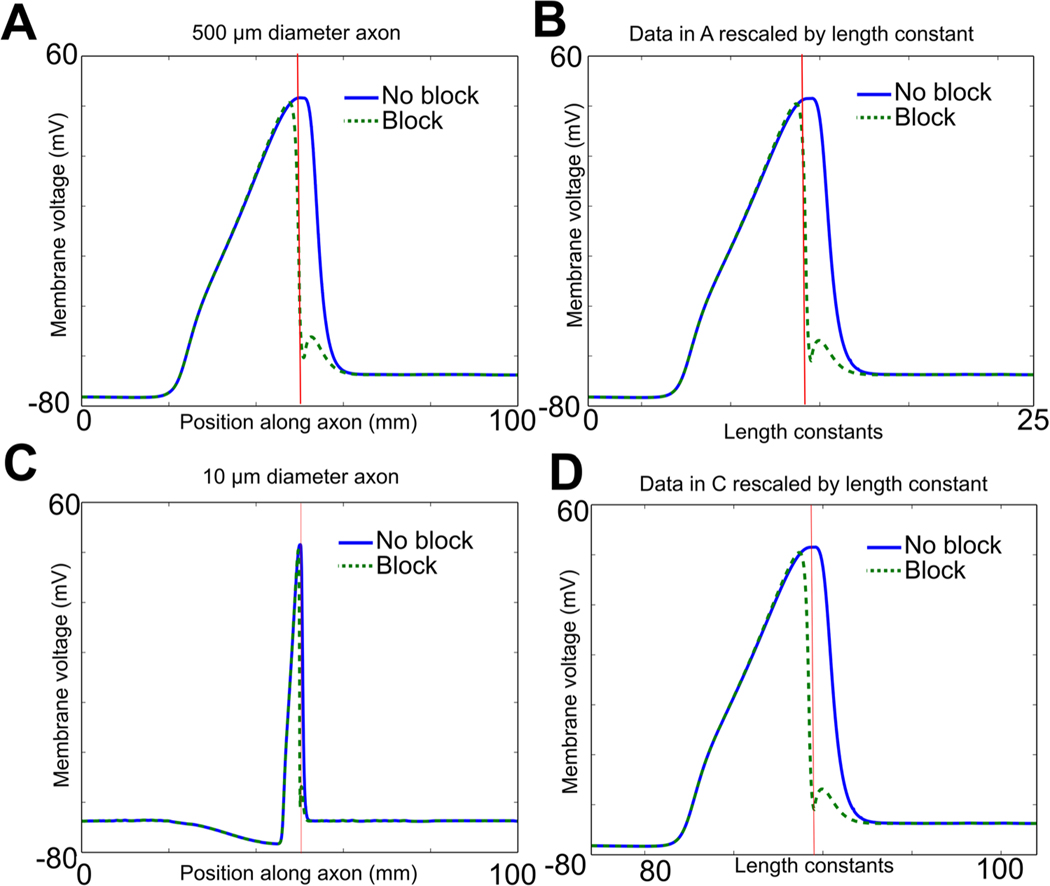
Spread of effects of block currents beyond the region of elevated temperature in the model squid giant axon scales with diameter. (A) At the time that the peak of the action potential reached the middle of aa 500 *μ*m diameter axon at its original temperature in response to a stimulating current (2000 nA for 1 ms), the voltage along the entire axon was plotted (dark blue line). The action potential occupies a large fraction of the entire length of the axon. The action potential was again induced as the temperature of the central region was increased to 29.5 °C, and at the identical time, the voltage along the entire axon was plotted (dashed green line). Note that the voltage of the second action potential is identical to the first at the beginning and at the end of the axon. Anterior to the region of block, however, the voltage of the second action potential begins to significantly change relative to the voltage of the first action potential; these changes continue beyond the region of elevated temperature as well. The vertical red line indicates the region of block. (B) Effect of spread of block currents rescaled by the axonal length constant. Data in panel (A) were rescaled by the length constant of the original axon (see text). The vertical red line indicates the region of block. (C) Effect of spread of block currents beyond the region of elevated temperature in a smaller unmyelinated axon. The simulations done in panel (A) were repeated in a 10 *μ*m diameter axon. The effect of the block currents clearly extends beyond the region of elevated temperature. Vertical red line indicates region of block. (D) Effect of spread of block currents rescaled by the length constant. Data in panel (C) were rescaled by the length constant of the original 10 *μ*m axon (see text). After rescaling, results in the 10 *μ*m diameter axon are essentially identical to those in the 500 *μ*m diameter axon (compare panels (B) and (D)). Vertical line indicates region of block.

To determine the spatial extent of an action potential in the absence of block, an action potential was initiated in response to a stimulating current (2000 nA for 1 ms) in an axon at its original temperature. At the instant the peak of the action potential reached the middle of the axon, we recorded the voltage along the entire axon (figure 6(A), dark blue line). Note the large spatial extent of the action potential. It is also worth noting the difference in shape from the action potentials shown in figure 4: in that figure, we record the change in voltage over time at the initial point of the block region and at the end of the axon, so we look at the time profile at a *single point in space*. In contrast, here we record the change in voltage over the entire length of the axon (i.e. over all space) at a *single instant in time*. To determine the effect of the spatial extent of the thermal block on the action potential, an action potential was initiated while the minimum length along the axon necessary for thermal block (1.12 mm) had its temperature increased to 29.5 °C, starting at the middle of the axon. At the identical time after the stimulating current initiated the action potential, we recorded the voltage along the entire axon. Note the large spatial extent of the block (figure 6(A), dashed green line). We rescaled these results by the axonal length constant so they could be compared to results obtained in axons of different diameters (figure 6(B)). We then repeated the simulation using a 10 *μ*m diameter axon (figure 6(C)); when the results were rescaled by the length constant, they were essentially identical to those observed in the 500 *μ*m diameter axon (figure 6(D); compare figure 6(B)).

**Figure 7.**
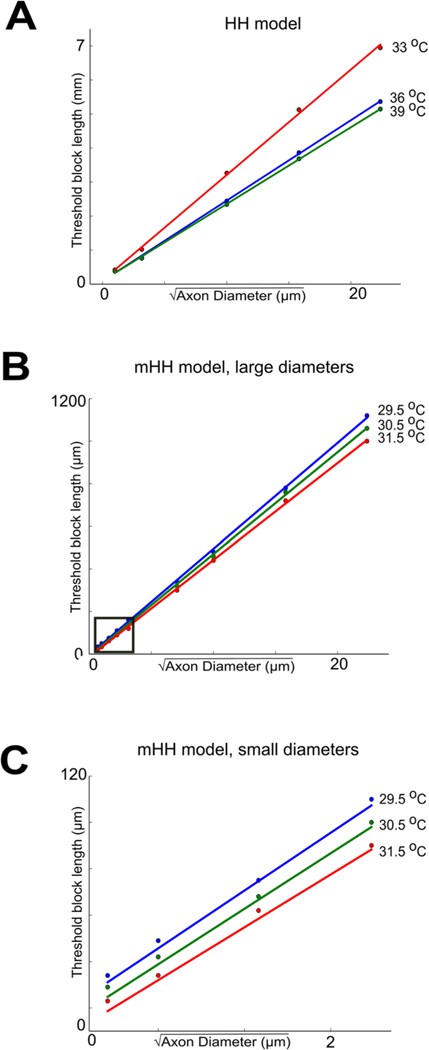
Thermal block length scales with the square root of axon diameter. (A) Thermal block length scales linearly with the square root of axon diameter in the unmodified Hodgkin/Huxley model, and length decreases with increasing temperature. (B) Thermal block length scales linearly with the square root of axon diameter in the modified Hodgkin/Huxley model, and the minimum length required for thermal block decreases with increasing temperature. (C) Expanded view of region highlighted by a square in panel (B). The linear relationship between the square root of axon diameter and threshold block length is observed for axon diameters similar to those of vertebrate unmyelinated C fibers.

The scaling of thermal block suggested that it would act first on smaller-diameter axons, rather than on larger-diameter axons. Once axons are scaled by the square root of their diameters, the spatial spread of voltage at a fixed point of time becomes identical. In turn, this implies that the voltage changes experienced by the voltage-dependent ion channels will also be identical, and thus the currents induced by these depolarizations will be identical. As a consequence, this numerically confirms the mathematical model of Lothet et al (2017) (supplemental material), which demonstrated that the length of block would scale with the square root of axon diameter. In contrast, extracellular currents are known to act on larger-diameter axons before smaller-diameter axons, since these currents scale as the square of the axon diameter (Rattay 1986). To further test the predictions of the mathematical analysis (Lothet et al 2017), we determined the minimum lengths of the central region that needed to be increased in temperature to induce thermal block as a function of axon diameter. As predicted by the mathematical model, the block lengths scaled linearly with the square root of the axon diameter (figure 7). The scaling effects were observed for the Hodgkin/Huxley model (figure 7(A)), as well as for the modified Hodgkin/Huxley model (figure 7(B)) even at diameters that are similar to those of unmyelinated C fibers in vertebrates (figure 7(C)). Furthermore, as temperatures were increased, the minimum block length decreased for both models (figure 7).

### Action potential initiation can be thermally blocked

**Figure 8.**
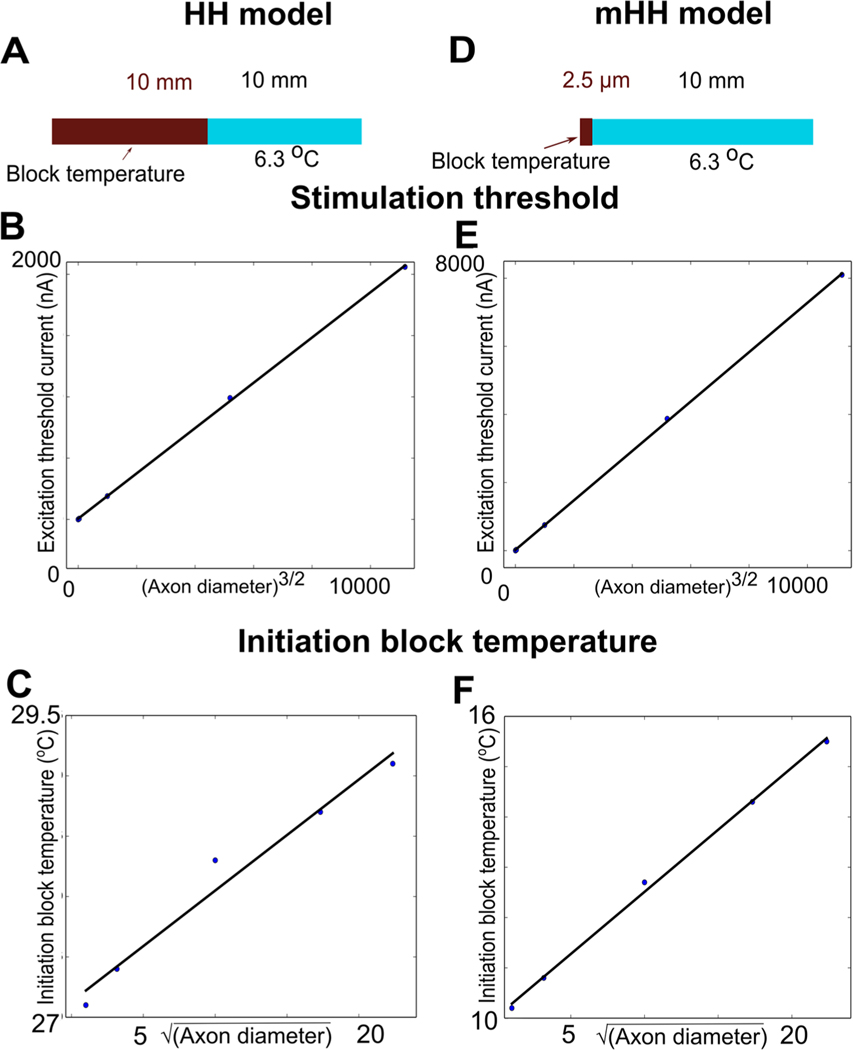
Scaling of thermal block of action potential initiation. (A) Schematic of the model axon for the Hodgkin/Huxley model, indicating the initial region (10 mm) that was used to determine the minimum current needed to initiate an action potential at the control temperature (6.3 °C) and then subjected to an increase in temperature to determine the minimum temperature to block action potential initiation. (B) Scaling of minimum current needed to initiate an action potential in the Hodgkin Huxley model. Minimum current (pulse length: 0.5 ms) was determined for axons whose diameter was 1 *μ*m, 10 *μ*m, 100 *μ*m, 300 *μ*m and 500 *μ*m. The minimum current scales with the axon diameter raised to the 3/2 power. See text. *R*^2^ = 0.99. (C) The minimum temperature needed to block action potential initiation using the threshold current determined for each axon diameter in the Hodgkin/Huxley model scales linearly with the square root of the axon diameter. *R*^2^ = 0.97. (D) Schematic for initial increase in temperature of modified Hodgkin/Huxley model. Because the potassium currents generated by the model are much larger, a smaller region of the axon can be subjected to an increase in temperature (2.5 *μ*m). (E) Minimum current for inducing an action potential again scales with the axon diameter raised to the 3/2 power. *R*^2^ = 0.99. (F) Minimum temperature for block when action potentials are initiated by the appropriate threshold current scales linearly with the square root of axon diameter. *R*^2^ = 0.97.

When an action potential is initiated in the initial segment of the axon, the currents generated are larger than those generated by the propagating action potential (Kole et al 2008). Since the thermal blocks described above were applied to a propagating action potential, thermal block of action potential initiation might be different (schematics of experiment shown figures 8(A) and (D), respectively). To determine how thermal block of initiation scales with axon diameter, we first determined the minimum current needed to initiate an action potential for a given diameter axon. Whenever an action potential is initiated using a very short current pulse, there is little time for activation of voltage-dependent ion channels, and thus the current needed to reach the voltage threshold is inversely proportional to the passive input resistance of the cable. In turn, this implies that the minimum current needed to initiate an action potential would scale as the square root of the axon diameter cubed (Jack et al 1975, p 32). Indeed, the minimum currents needed to initiate an action potential scaled in this way for both the Hodgkin Huxley and the modified model (figures 8(B) and (E), respectively). When thermal block was applied to either model after it was stimulated by the appropriate threshold current to initiate an action potential, the inhibition block temperature again scaled with the square root of axon diameter (figures 8(C) and (F), respectively).

These results suggest that thermal block in unmyelinated axons, especially at higher temperatures, are primarily due to the activation of voltage-dependent potassium ion channels in response to depolarization, which induce a strong hyperpolarization and thus block action potential initiation and propagation, and can act preferentially to block smaller-diameter axons before larger-diameter axons.

## Discussion and conclusions

To our knowledge, this is the first study to provide quantitative evidence for a mechanism of thermal block that was originally suggested by Huxley (1959): thermal block at higher temperatures is due primarily to activation of voltage-dependent potassium ion channels (figure 4). It is also the first model to capture the changes in the rates of rise and fall of action potentials in the squid giant axon based on the experimental data of Rosenthal and Bezanilla (2000, 2002). Thermal block also appears to work for repetitive firing as well as for single action potentials (figure 5). In our previous study (Lothet et al 2017, figures 2, 3 and supplemental figure 5), we demonstrated that thermal inhibition could block a repetitively stimulated compound action potential. The results shown in figure 5 suggest that the mechanism of this block is the same as the mechanism that blocks a single propagating action potential.

The data presented in this paper also provide further support for the prediction of a mathematical model based on the cable equation (Lothet et al 2017—supplemental materials) that treatments that act on surface ion channels (such as infrared light or drugs) will have effects that scale as the square root of the diameter of the axon (figures 6–8). In turn, this implies that small-diameter axons will have lower thresholds for responding to such treatments than will large-diameter axons. Since extracellular electrical currents, a major modality for affecting neural activity, preferentially affect large-diameter axons before affecting small-diameter axons (Rattay 1986), the results presented in this study open up a range of new approaches to analyzing nervous system function, and new ways to alter physiology or to treat diseases that selectively affect small-diameter unmyelinated axons (Dubin and Patapoutian 2010, Duchesne et al 2016, Pekala et al 2016).

The modified Hodgkin/Huxley model presented in this paper more accurately describes the response of squid giant axons to changes in temperature than does the original Hodgkin Huxley model (figure 1). The modified model still has significant limitations. It does not fully capture the complexities of the activation and inactivation of the sodium ion channel (e.g. Clay (1998) and Sangrey et al (2004)), which may be important for understanding the response of axons to increasing temperatures. It does not capture the sub-classes of voltage-dependent potassium ion channels that have been described in the squid giant axon (Rosenthal and Seeburg 2012), which could account for some aspects of temperature responses (Garrett and Rosenthal 2012). Finally, if the modified model were to be applied to studies of unmyelinated C fibers in vertebrates, it would also need to capture the properties of other ion channels that are known to be present and sensitive to temperature, such as TRP channels (Patapoutian et al 2003, Cortright et al 2007, Voets 2014). Despite these limitations, both the original and the modified Hodgkin/Huxley model are in substantial qualitative agreement, and the results presented in this study are an important step towards developing a fuller understanding of a novel modality for modifying neural activity.

Previous studies of invertebrate axons have indicated that the operating temperatures of poikilotherms (such as fish, lizards, insects, or mollusks) range from above freezing to about 40 °C, and that animals can tolerate changes in temperature over this range both through changes in behavior and in properties of their neurons (Prosser and Nelson 1981, Janssen 1992, Galarza-Muñoz et al 2011, Robertson and Money 2012). In contrast, the operating temperatures for homeotherms is about the top of the range for poikilotherms, and homeotherms can generally tolerate only a few degrees of change in temperature. Elevated temperatures can block unmyelinated fibers in the gray matter of mammalian cerebellum and hippocampus, which may contribute to many of the symptoms of fever (Pekala et al 2016). In the periphery, temperature can significantly affect the transmission of motor signals in patients suffering from de-myelinating diseases such as multiple sclerosis (Schauf and Davis 1974), as well as in normal subjects (Rutkove et al 1997). Thus, applying the results of these studies to vertebrate axons will require the development of models more suitable to vertebrate operating temperatures.

The model we have presented has implications for understanding thermal block in mammalian systems. Previous studies in the vagus nerve of the musk shrew *Suncus murinus* have shown that *unmyelinated* small-diameter axons are more susceptible to inhibition due to infrared laser light than are large-diameter axons (Lothet et al 2017), consistent with the predictions of the mathematical model in that paper, and the numerical model in this paper. Studies of individual *unmyelinated* C fibers in primates that are sensitive to both temperature and mechanical stimuli suggested that fatigue in response to high temperature stimuli, which increased strongly with temperature, might be due to prolonged hyperpolarization; the authors point out, however, that transduction of the signals and initiation of action potentials might also play an important role (Peng et al 2003). A recent study in *myelinated* peripheral human sensory and motor axons demonstrated that safety factors were reduced in response to hyperthermia, which could lead to thermal block, and modeling of the results suggested that a major factor underlying these changes was alterations in slow potassium ion channels. In addition, a hyperpolarizing activated cation current, $I_{h}$, was reduced, and this could also reduce recovery of axons from hyperpolarization due to activity (Howells et al 2013). As the investigators point out, the sensitivity of these myelinated fibers to temperature has significant implications for their responses to fever and to demyelinating diseases such as multiple sclerosis.

To generalize these results to vertebrate unmyelinated axons, it will also be critical to characterize the role of the other voltage-activated channels that have been found in these axons, such as TRP channels, many of which are thermally sensitive (Patapoutian et al 2003, Cortright et al 2007, Voets 2014). In very fine axons, in which there are many fewer ion channels, it may be necessary to modify the kinetics of ion channels, which differ across species (Krouchev et al 2015), as well as incorporate channel noise and the non-uniform distributions of ion channels along the axon (Neishabouri and Aldo Faisal 2014). Capturing the changes in the resting potential, which could affect the voltage-activated channels, may require incorporating the temperature-dependence of the chloride channels (Pusch et al 1997). If the constricted extracellular space affects the external resistance, the model could also be modified to incorporate the effect of this resistance (Wu and Wikswo 1997).

Other investigators have begun to create models of the effects of infrared laser light on neural excitability. To model the excitatory effects of infrared light, a model modified the capacitance of the Hodgkin/Huxley model in response to increasing temperature. The resulting model generated action potentials in response to rapid temperature increases (Fribance et al 2016). The mechanisms, targets and progress in modeling thermal excitation has been recently reviewed in Shapiro et al (2012), Thompson et al (2014), Zhao et al (2016), Plaskin et al (2017) and Ford et al (2018). A modeling study of *Xenopus myelinated* fibers indicated that high temperatures from a continuous wave laser focused on single nodal regions could block action potential initiation or propagation, which they argued was consistent with faster sodium inactivation and stronger potassium channel activation. These investigators also showed, experimentally, that electrical stimulation thresholds to evoke an electrical response increased in response to laser heating (Mou et al 2012).

Developing better models of the effects of laser light, and other thermal modalities for affecting neural activity, may provide important insights into the relationship between the spatio-temporal dynamics of temperature and neuronal function during disease. In the periphery, temperature can significantly affect the transmission of motor signals in patients suffering from de-myelinating diseases such as multiple sclerosis (Schauf and Davis 1974), as well as in normal subjects (Rutkove et al 1997).

Finally, these results suggest that the predictions of the mathematical model (Lothet et al 2017—supplemental materials) that smaller-diameter axons may have lower thresholds for response than larger-diameter axons to any modality that acts along their surface. Thus, cuff applications of ion channel blockers or agonists may selectively affect the sub-population of smaller-diameter axons, and this in turn could lead to new approaches to analyzing the nervous system, or to novel treatments of diseases that affect smaller-diameter axons.

In conclusion, a range of models of thermally-sensitive unmyelinated axons suggest that thermal inhibition, especially at higher temperatures, is due to the faster activation of voltage-dependent potassium ion channels. In turn, these channels generate a hyperpolarizing current that effectively blocks depolarizing currents. The minimum length needed to block action potential initiation, propagation, or repetitive firing scales with the square root of an axon’s diameter. Manipulations of relatively short axonal regions containing voltage-dependent potassium ion channels could selectively block fine, unmyelinated axons, such as C fibers, that carry pain and other sensory information.

## Supplementary Material

SupplementaryFigures
