# Supplementary material for: Thermal block of action potentials is primarily due to voltage-dependent potassium currents: a modeling study
Source: J Neural Eng. Author manuscript; Available in PMC 2024 Jun 21. (PMC11190670; doi:10.1088/1741-2552/ab131b)
Supplement: SupplementaryFigures [file NIHMS1991942-supplement-SupplementaryFigures.docx]

Supplementary Figure 1


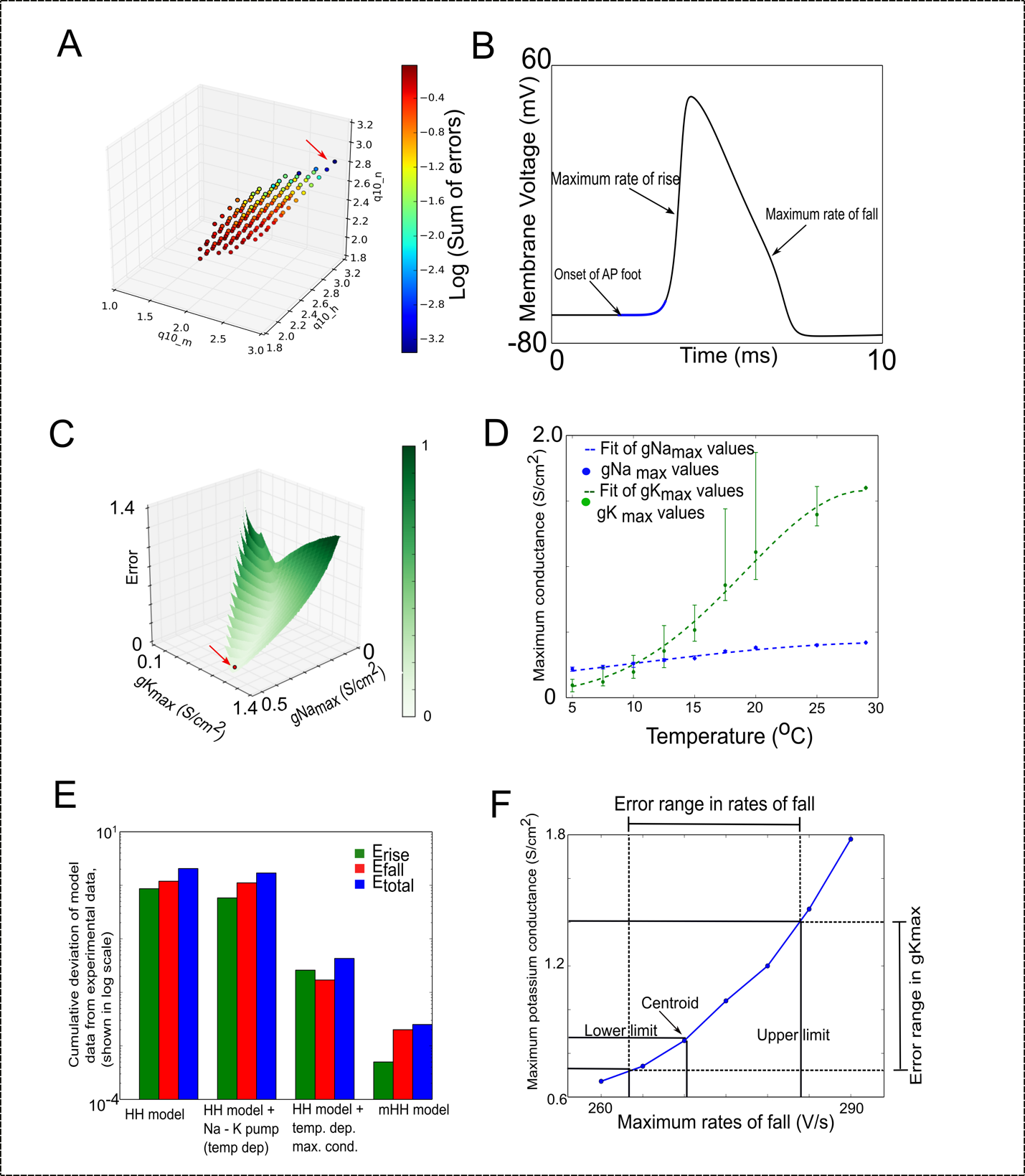


**Supplementary Figure 1.** Temperature-dependent modifications of the original Hodgkin Huxley model. **A**. To find the valid set of Q_10_ values for all three gates (*m*, *n*, *h*), we performed an exhaustive three-dimensional grid search to arrive at the correct Q_10_ parameter. At a specific temperature, the Q_10_ values for each gate (*m*, *n*, and *h*) were systematically varied from 1.0 (gates not temperature dependent) to 3.0 (the value in the Hodgkin/Huxley model) in increments of 0.1, totaling 20*20*20 = 8,000 cases. In each of these cases, the total amplitude of the action potential was evaluated, if an action potential was generated. We defined the total amplitude as the absolute difference between the resting potential and the peak of the action potential. These total amplitudes were then compared against the experimentally reported values obtained at that specific temperature (Hodgkin and Katz, 1949). We focused on the set of Q_10_ values that produced action potentials whose total amplitudes were within 5% of the reported values. Among the selected sets of values, we evaluated the errors for maximum rates of rise and fall, using equations (11) – (13). The combined errors for rates of rise and fall of action potentials are plotted. The Q_10_ values for each of the gates are shown along the three axes. Each dot in the figure represents a set of Q_10_ values that produce an action potential whose peak amplitude is within 5% of the experimentally reported values. The color of each dot represents the sum of the errors for the particular set of Q_10_ values. The range of error values are shown by the adjoining color bar (using a logarithmic scale). As the color of the dot changes from red to blue, the error decreases. The Q_10_ values associated with the least error (shown with a red arrow) were chosen for the particular temperatures. The figure shows the analysis for 5˚C. Similar analyses were done for 10˚C, 15˚C, 20˚C and 25˚C. **B**. Measurements from the model action potential that were compared to the data of Rosenthal & Bezanilla (2000). The model action potential shown here was generated by the modified Hodgkin/Huxley model at 5˚C. The maximum rate of rise, the maximum rate of fall, and the onset of the foot of the action potential are all indicated. See Methods section for how these were used for parameter estimation. **C**. To find the sodium and potassium maximum conductance that fit the maximum rates of rise and fall of action potential recorded experimentally, we performed an exhaustive grid search by varying the maximum sodium conductance from 0.12 S/cm^2^ (original value in the Hodgkin/Huxley model) to 0.5 S/cm^2^, and the potassium maximum conductance from 0.036 S/cm^2^ (original value in the Hodgkin/Huxley model) to 1.5 S/cm^2^, in increments of 0.02 S/cm^2^. Errors were calculated at each point of these values. The expression used to calculate the errors between the experimentally reported maximum rates and the simulation-derived rates are described in equations (11) – (13) in the Methods. For a particular pair of maximum sodium and maximum potassium conductances, the sum of the errors observed for the maximum rates of rise and maximum rates of fall are plotted (plot shows the results obtained from searches at 25^˚^C). The sum of the errors smoothly decreases as the sodium and potassium conductance increase without any local minima or maxima. At points with low values of sodium maximum conductance and high values of potassium conductance, an action potential was not generated, and hence the errors for those value pairs are not shown in the plot. The red arrow shows the point where the sum of the errors reached the global minimum. We used the sodium and potassium conductance obtained at this point as the basis for the peak sodium and potassium conductances in the modified Hodgkin/Huxley model. This analysis was repeated for all the temperatures reported in the experimentally published work, which ranged from 5^˚^C to 25^˚^C. **D**. Variation of the peak sodium (blue) and potassium (green) conductances with temperature (equation (8)). The R^2^ value for the Gaussian fits is > 0.99. See the text for details. **E**. Errors in rates of rise (E_rise_, green), rates of fall (E_fall_, red) and total error (E_total_, blue) plotted on a logarithmic scale for three versions of the model (equations (11) - (13)). The errors associated with the unmodified Hodgkin/Huxley model are shown in the left-most group of bars; the errors associated with the Hodgkin/Huxley model to which a temperature-dependent Na/K pump were added are shown in the next group of bars; the errors associated with the Hodgkin/Huxley model and temperature-varying peak conductances are shown in the next group of bars, and the errors associated with the Hodgkin/Huxley model which has both a temperature-dependent sodium-potassium pump and the modified peak conductances are shown in the right-most bars (we refer to this as the modified Hodgkin/Huxley model, or the mHH model). The mHH has the smallest errors. **F**. Translating errors in the original data (rate of fall at 17.5˚C) into error ranges for the estimated model parameters. See Methods for details.

Supplementary Figure 2


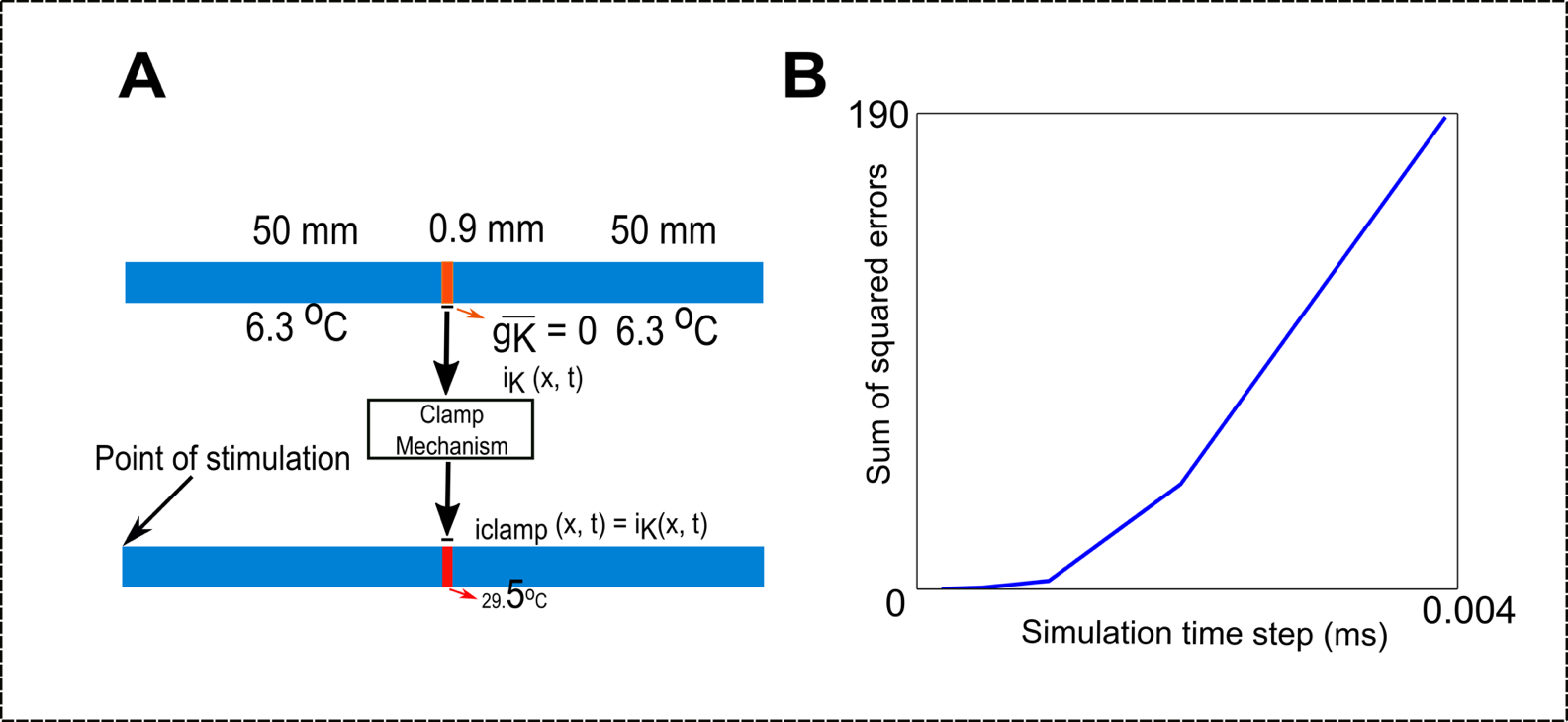


**Supplementary Figure 2.** Injecting compensatory current. **A**. Schematic illustration of the compensatory current experiment. (Top schematic) The currents were measured from a model neuron whose voltage-dependent potassium ion channel conductances were maintained at their control values (indicated by orange rectangle), and whose temperature was at 6.3˚C throughout. (Bottom schematic) The opposite current was injected into each of the corresponding segments of the second axon whose peak potassium conductances were set to zero (indicated by the red rectangle) to ensure that the second axon maintained its original resting potential despite the removal of the voltage-dependent potassium ion channels (see Supplementary Figure 3H for the potential change in the central region with and without compensatory current). **B**. Mean square error in change in voltage along the length of an axon in the presence of compensatory current compared to a control axon as a function of integration step size. See Methods.

Supplementary Figure 3


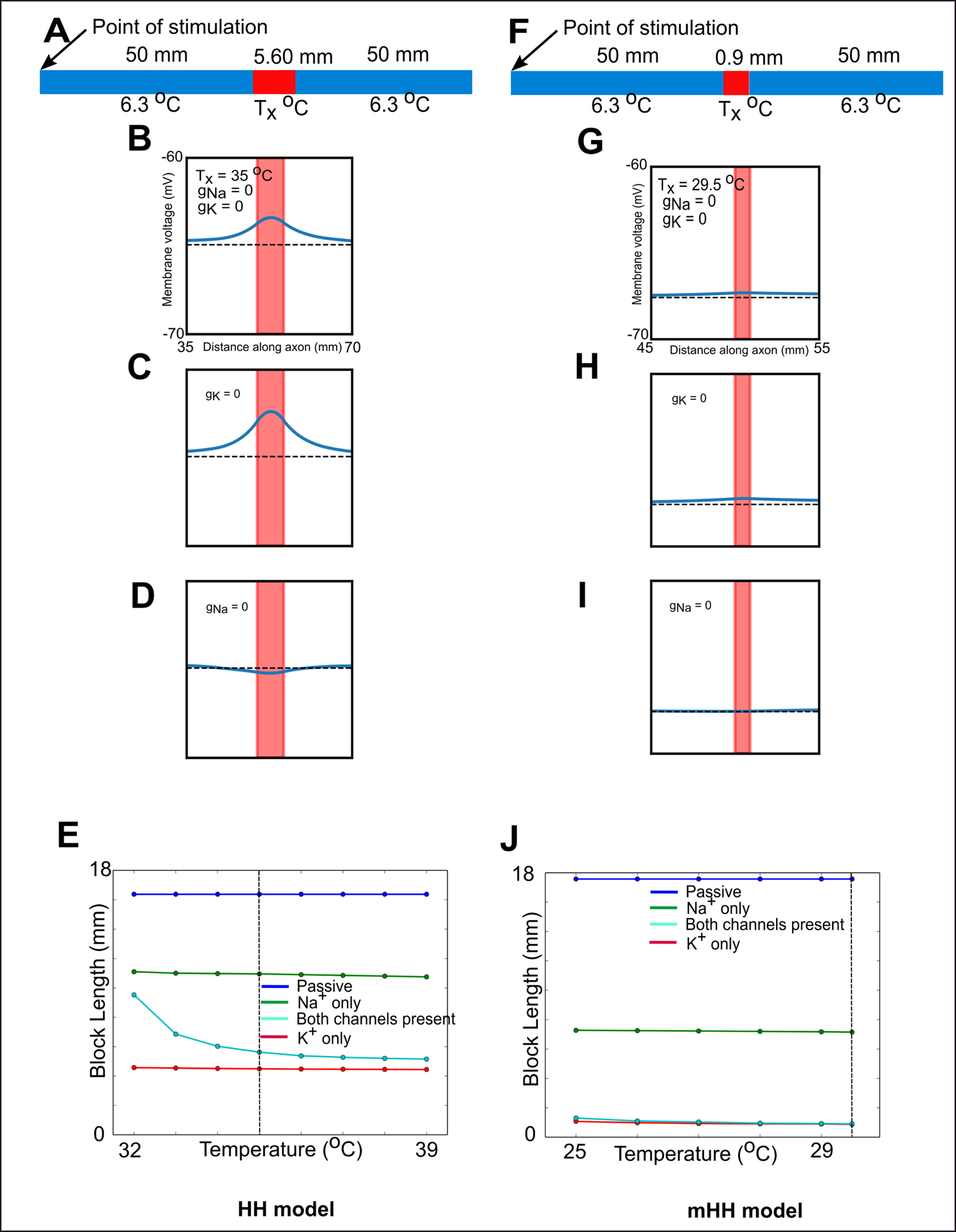


**Supplementary Figure 3**. Small changes in membrane potential due to removal of ion channels do not significantly alter the minimal lengths necessary to block action potential propagation, and still demonstrate that voltage-gated ion channels, especially potassium ion channels, are critical for thermal block. In addition, thermal block for single action potentials is sufficient to block repetitive firing. **A**, **F**. Schematics of the experiments for the Hodgkin/Huxley model and the modified Hodgkin/Huxley model, respectively. An action potential was initiated at the left side with an injected current pulse (2000 nA, 1 ms). Ion channel conductances were altered, and the central region of the model axon was subjected to an increase in temperature. The minimum length needed to block the conduction of the action potential was determined. Unlike the data in Figure 4, compensatory current was not used to ensure that the resting potential was kept at its original value (-65 mV) after the ion channel conductances were manipulated. **B**, **G**. Setting the conductances of both the sodium and potassium voltage-gated ion channels to zero induces a depolarization of the membrane towards the leak conductance’s equilibrium potential in both the Hodgkin/Huxley (**B**) and modified Hodgkin/Huxley (**G**) models. **C**, **H**. Setting the conductance of the voltage-gated potassium ion channels to zero induces a depolarization of the membrane in both the Hodgkin/Huxley (**C**) and modified Hodgkin/Huxley (**H**) models. **D**, **I**. Setting the conductance of the voltage-gated sodium ion channels to zero induces a hyperpolarization of the membrane in both the Hodgkin/Huxley (**D**) and modified Hodgkin/Huxley (**I**) models. **E**, **J**. Despite the small changes in potential when ion channel conductances are eliminated, the lengths necessary to block action potential conduction are dominated by the voltage-gated potassium ion channels for both models, especially at higher temperatures. At the lowest block temperatures, the Hodgkin/Huxley model (**E**) is also significantly affected by the block lengths of the voltage-gated sodium ion channels.
